# Design, synthesis and anticancer evaluation of naphthalen-1-yloxyacetamide derivatives against MCF-7 cells

**DOI:** 10.1039/d5ra06524k

**Published:** 2025-09-30

**Authors:** Maha Ali Alghamdi, Mustafa R. Abdulbaqi, Rana Abdullah Alghamdi, Eman Fayad, Dalal Nasser Binjawhar, Hanadi A. Katouah, Abdullah Yahya Abdullah Alzahrani, Amal M. Youssef Moustafa

**Affiliations:** a Department of Biotechnology, College of Sciences, Taif University P.O. Box 11099 Taif 21944 Saudi Arabia; b Department of Pharmaceutics, College of Pharmacy, Al-Naji University Baghdad 10015 Iraq; c Department of Chemistry, Science and Arts College, King Abdulaziz University Rabigh Saudi Arabia; d Regenerative Medicine Unit, King Fahd Medical Research Centre, King Abdulaziz University Jeddah Saudi Arabia; e Department of Chemistry, College of Science, Princess Nourah bint Abdulrahman University P.O. Box 84428 Riyadh 11671 Saudi Arabia; f Chemistry Department, College of Science, Umm Al-Qura University 21955 Makkah Saudi Arabia; g Department of Chemistry, Faculty of Science, King Khalid University Abha 61413 Saudi Arabia; h Chemistry Department, Faculty of Science, Port Said University 42511 Port Said Egypt amalyoussef840@gmail.com a.moustafa@sci.psu.edu.eg

## Abstract

Herein, a set of naphthalen-1-yloxyacetamide-tethered 2,3-disubstituted acrylamide conjugates was synthesized in a good yield *via* the reaction of naphthalene-1-yloxyacetohydrazide and an equimolar amount of respective ethyl 2,3-disubstituted acrylate ester in pure ethanol with a catalytic base of fused sodium acetate. All conjugates were assessed for their anti-proliferative activity against the MCF-7 *Br Ca* cell line. The results found that conjugate 5d with 3-(4-methoxyphenyl)-2-phenylacrylamide attached to the naphthalen-1-yloxyacetamide moiety demonstrated potent cytotoxic activity against the investigated MCF-7 *Br Ca* cells. Thereafter, compounds 5c, 5d, and 5e were subsequently evaluated for their aromatase inhibitory activity. Moreover, the representative promising conjugate in cytotoxic and aromatase inhibition assays was tested to investigate its impact on the cell cycle analysis and apoptosis promotion with the aim to get more insight into the antitumor mechanism of action of the target naphthalen-1-yloxyacetamide-acrylamide conjugates. In the examined MCF-7 cells, compound 5d heightened cell cycle arrest during the G1 phase and provoked cellular apoptosis. Furthermore, compound 5d increased the apoptosis percentage *via* the expressive downregulation of the anti-apoptotic protein Bcl-2 and the upregulation of Bax and caspase 9 levels relative to untreated groups.

## Abstract 2

Herein, a set of naphthalen-1-yloxyacetamide-tethered 2,3-disubstituted acrylamide conjugates was synthesized in a good yield and evaluated for their cytotoxicity against MCF-7 breast cancer cells.

## Graphical abstract

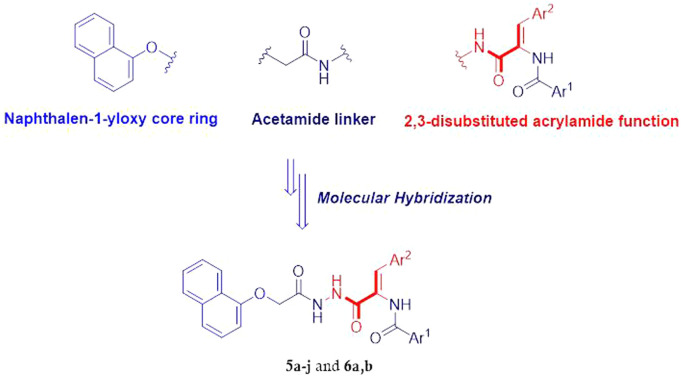

## 1. Introduction

Breast cancer (*Br Ca*) is the second most frequent cancer worldwide and the fifth leading cause of cancer-related death.^1^ It is the leading cause of cancer-related deaths among women in less developed regions, and the second leading incidence in more developed areas, behind lung cancer.^2^ High levels of estrogen have been noted to increase the risk of developing breast malignancy, especially in the estrogen receptor-positive (ER+) subtype, which is the subtype diagnosed in 70% of cases.^3^ It was reported that estrogen facilitates growth and proliferation of several *Br Ca* cell types (*i.e.*, estrogen-dependent mammary carcinoma cells (MCF-7)).^4^ Drugs that limit the synthesis of estrogens or inhibit estrogen receptor (ER) continue to be the mainstay of *Br Ca* treatment and management.^5,6^ Aromatase is expressed in a variety of tissues, including adipose tissues.^7^ Aromatase is a crucial enzyme in the biosynthesis of estrogens, which are hormones that aid in the development of some forms of *Br Ca*.^8^ The conversion of androgens into estrogens is catalyzed by aromatase.^9^ Given the importance of estrogen production in encouraging the growth of *Br Ca*, aromatase has emerged as critical target for the search for anticancer agents in the treatment of *Br Ca*.^10^ Aromatase inhibitors (AIs) are classified based on the difference between their chemical structures and mode of actions as steroidal and nonsteroidal AIs.^11^ Among them, nonsteroidal AIs (NSAIs) have received increased interest in clinical applications due to their reversible inhibitory nature.^12^ Among these, third generation NSAIs (*i.e.*; Letrozole, anastrozole and vorozole) were identified as the most effective treatments for estrogen-dependent postmenopausal women with advanced breast cancer.^12^ However, their notable adverse effects (*i.e.*, decreased bone density and increased risk of cardiovascular diseases) and potential drug resistance in prolonged usage remain difficult challenges to resolve.^13^ Accordingly, the development of novel NSAIs with improved potency and selectivity is still an active field of research in the modern medicinal chemistry.

Naphthalene nucleus is planar bicyclic molecule made up of two benzene rings that have fused together.^14^ According to Huckel's rule, it is the simplest polycyclic aromatic hydrocarbon containing 10π electrons in its structure.^15^ In medicinal chemistry, naphthalene scaffold constitute a versatile and adaptable platform.^16^ It has been reported that the naphthalene scaffold is a good surrogate for the benzene ring, improving the active compounds' chemical and metabolic stability while maintaining pharmacology.^17^ This scaffold's diverse biological activities resulting from structural modifications make it an attractive moiety in drug design advancement.^18^ Derivatives based on naphthalene have been shown to exhibit a variety of antagonistic properties, including anticancer, antimicrobial, antiviral and antitubercular agents.^19–22^ Naphthalene derivatives have been shown to exhibit anticancer properties through a variety of mechanistic pathways, like aromatase inhibitors, potent topoisomerase inhibitors and as microtubule polymerization inhibitors.^23–25^ For example, Furomollugin I is a naturally occurring naphthalene-based compound revealed outstanding anticancer properties.^26^ In addition, the naphthochalcone derivative Hymnpro II exhibited antiproliferative activity in several human solid tumor cell lines and suppressed xenograft tumor growth in nude mice.^27^ Additionally, the antiproliferative effect of naphthalen-1-yl linked pyrazole derivative III on MCF-7 cells was associated with cellular cycle arrest followed by apoptosis.^28^ Also, the bis-naphthalene-sulphonamide derivative IV was identified as aromatase inhibitors with IC_50_ value of 0.21 μM relative to reference standard Letrozole (IC_50_ = 0.19 μM) with high safety profile favouring cancer cells.^29^

**Fig. 1:**
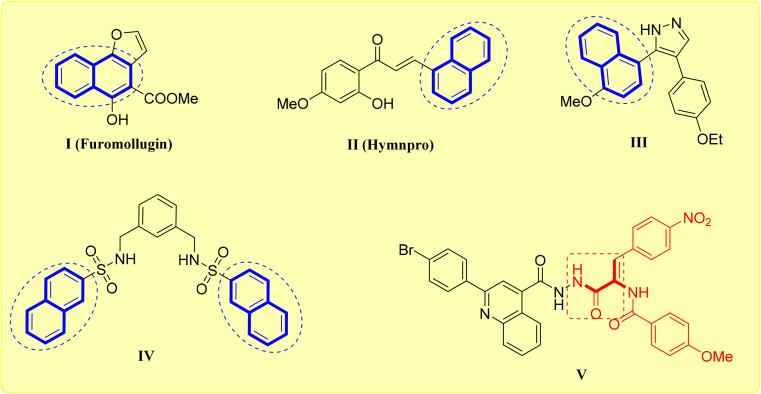
Naphthalene I–IV and 2,3-disubstituted acrylamide V derivatives as anticancer agents.

In the recent years, acrylamide scaffold is appreciated owing to their therapeutic properties which have been reported for certain derivatives especially in the treatment of *Br Ca* cells.^30^ The search on acrylamide-based covalent inhibitors has advanced significantly, showing promise in a number of therapeutic domains, especially as tubulin and kinase inhibitors for the management of different cancer types.^31,32^ An acrylamide fragments are very beneficial for joining arms with variously substituted aryl fragments to create side chain components.^33^ The structure of important moieties such as urea and hydrazone moieties was replaced with an acrylamide fragment that was comparable in length and structure to these important moieties, thereby ensuring the formation of crucial hydrogen bonding with the amino group in the target protein or receptor.^34,35^ 2,3-Disubstituted acrylamide-bearing derivative V (Fig. 1) explored the highest potency against MCF-7 breast cancer cells through its inhibitory effect on EGFR-TK.^36^

**Fig. 2:**
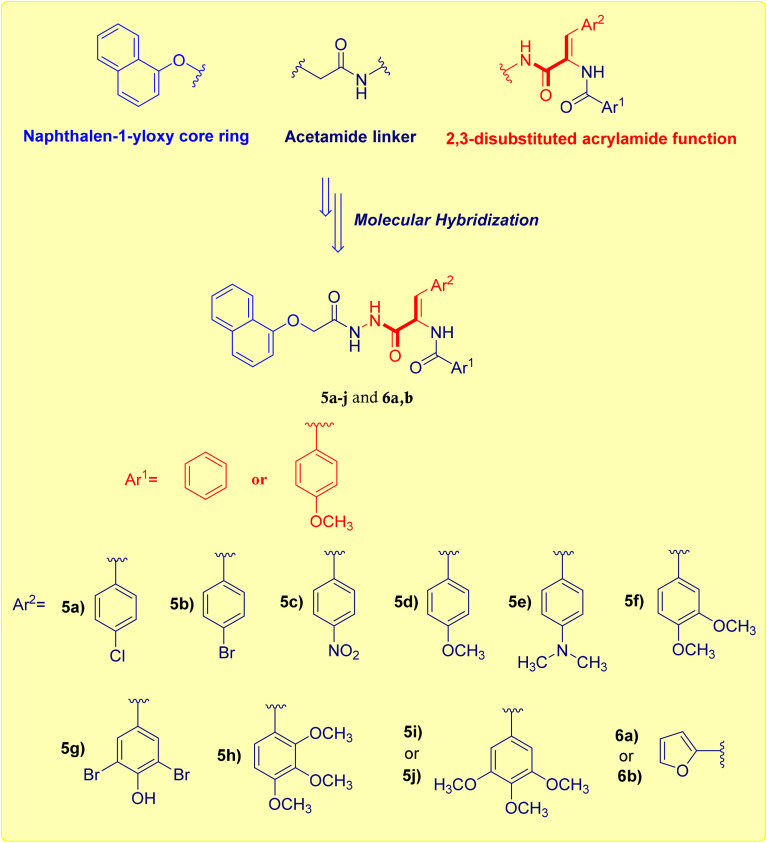
Design pathway adopted for naphthalen-1-yloxyacetamide-tethered acrylamide conjugates 5a–j and 6a, 6b.

**Scheme 1:**
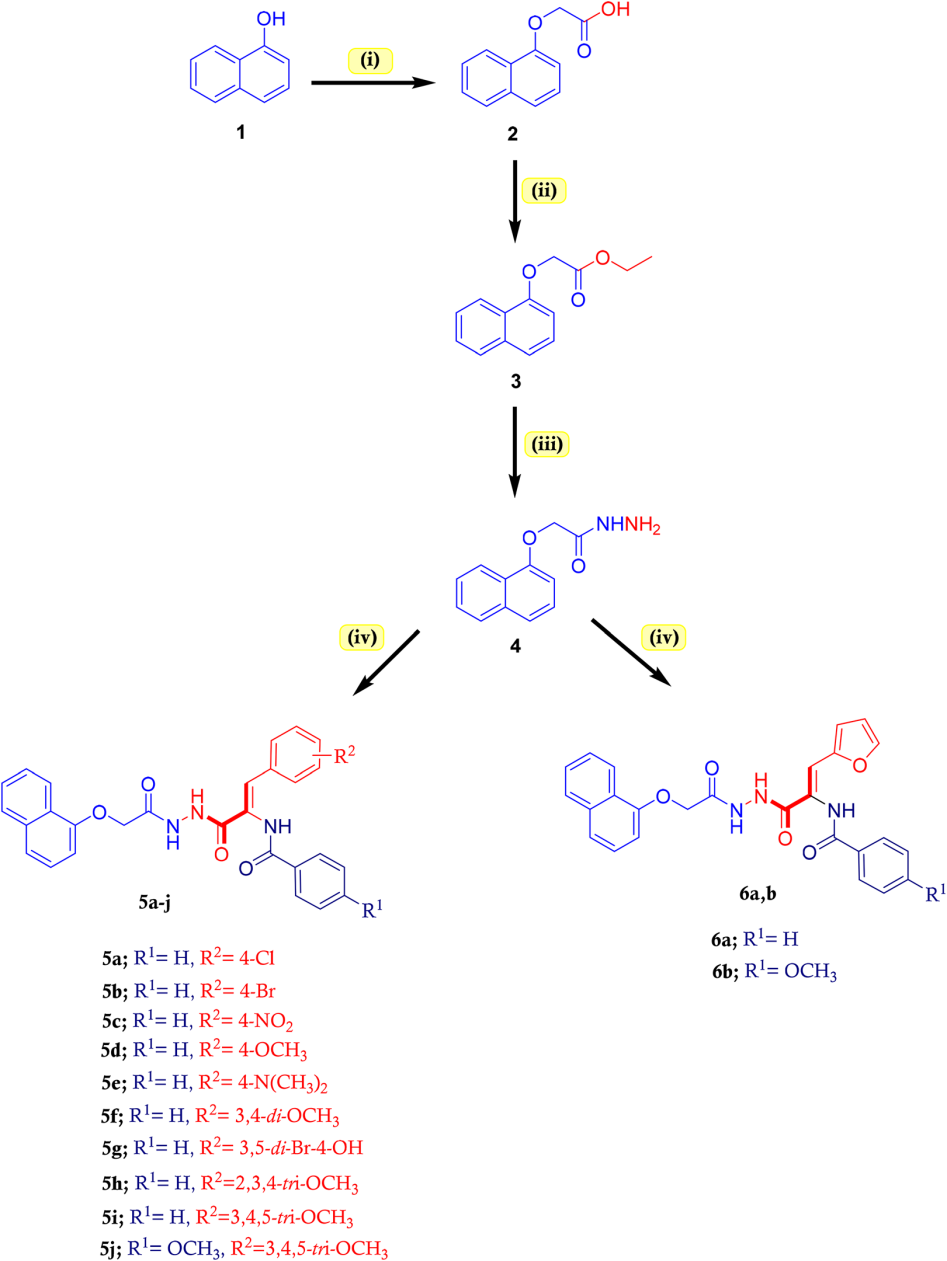
Synthesis of naphthalen-1-yloxyacetamide-tethered 2,3-disubstituited acrylamide conjugates 5a–j and 6a, 6b. Condition: (i) BrCH_2_COOH, K_2_CO_3_, acetone, reflux 12 h; (ii) pure EtOH, AcOH, reflux 16 h; (iii) NH_2_NH_2_·H_2_O, EtOH, reflux 8 h; (iv) respective ethyl 2,3-disubstituted acrylate ester, NaOAc, EtOH, reflux 12–14 h.

Based on the above reported studies we understood the potential of naphthalene and acrylamide conjugates in the treatment of cancer. In this context, the synthesis of a new series of naphthalen-1-yloxyacetamide-tethered acrylamide conjugates was reported (Fig. 2). The target conjugates were created from the starting material, 1-naphthol following the general pathway reported in Scheme 1. All the created conjugates were confirmed by various methods of spectral analysis such as ^1^H-NMR and ^13^C-NMR. The newly synthesized compounds were evaluated for their cytotoxicity against MCF-7 *Br Ca* cell line to investigate their potential as chemotherapeutic agents. The most active naphthalen-1-yloxyacetamide-tethered acrylamide conjugate 5d was subjected to cytofluorimetric analysis and aromatase inhibitory activity to explore the mechanism of cellular death. Moreover, the ability of compound 5d to induce apoptosis/necrosis in MCF-7 *Br Ca* cells was determined. Furthermore, its impact on the protein expression levels of apoptotic markers was measured.

## 2. Results and discussion

### 2.1. Chemistry

The synthetic route of the designed naphthalen-1-yloxyacetamide tethered acrylamide conjugates 5a–j and 6a, 6b were presented in Scheme 1. The target conjugates were prepared from the root starting material; naphthalen-1-yloxy acetic acid 2 which was achieved from the low-cost 1-naphthol and 2-bromoacetic acid *via* classical nucleophilic substitution reaction of phenolic OH group with alkyl bromide group.^37^ Ethyl naphthalen-1-yloxy acetate 3 was synthesized *via* classical condensation reaction by refluxing the naphthalen-1-yloxy acetic acid 2 with pure ethanol in the presence of catalytic concentrated sulphuric acid.^38^ Adopting condensation reaction of 3 with hydrazine hydrate in pure ethanol gave the key intermediate, naphthalen-1-yloxy acetic acid hydrazide 4.^39^ The formation of novel naphthalen-1-yloxyacetamide tethered 2,3-disubstituted acrylamide conjugates 5a–j and 6a, 6b was accomplished *via* the reaction of equimolar amounts of naphthalen-1-yloxy acetic acid hydrazide 4 with respective ethyl 2,3-disubstituted acrylate ester in refluxing pure ethanol with a catalytic base of fused sodium acetate. The structures of new conjugates were confirmed by elemental analysis and spectral data. Both the analytical and spectral data (^1^H-NMR and ^13^C-NMR) of newly synthesized naphthalen-1-yloxyacetamide tethered acrylamide conjugates 5a–j and 6a, 6b in DMSO-*d*_6_ were completely in accordance with the expected structures as indicated in the SI. ^1^H-NMR spectrum of naphthalen-1-yloxyacetamide tethered 2-(benzamido)-3-(4-methoxyphenyl)acrylamide 5d, as example, demonstrated the presence of three singlets in the range at *δ* 10.33–9.89 ppm integrated one proton each corresponding to three amidic NH protons. The multiplet signals in the aromatic region at *δ* 8.44–6.97 were ascribed to 1-naphthyl, phenyl and olefinic protons. The ^1^H-NMR spectrum also exhibited the characteristic signals for the methylene and methoxy protons. These proton signals were observed at *δ* 4.86 and 3.76 ppm, respectively. The ^13^C-NMR spectrum of conjugate 5d disclosed characteristic signals resonating around *δ* 160.19–106.21 ppm that were attributed to 1-naphthyl, phenyl and acrylate carbons. The three amide carbonyl (C=O) functionalities displayed in the ^13^C-NMR spectrum at the *δ* 167.11–164.96 ppm. The presence of carbon peaks at 66.98 ppm confirms the linkage of methylene group with acrylamide moiety. In addition to a resonance peak at *δ* 55.68 ppm was attributed to the methoxy carbon confirming the proposed structure.

### 2.2. Biological evaluation

#### 2.2.1. Cytotoxic activity against MCF-7 *Br Ca* cells

To assess the antiproliferative effects of the target naphthalen-1-yloxyacetamide tethered acrylamide conjugates 5a–j and 6a, 6b, a colorimetric MTT technique was utilized against MCF-7 *Br Ca* cell line.^40^ The results were listed as an IC_50_ values with antitumor drugs Doxorubicin (Dox) and Tamoxifen (Tam) were included as reference drugs in this study. As demonstrated in Table 1, the majority of the investigated naphthalen-1-yloxyacetamide tethered acrylamide conjugates revealed good cytotoxicity with IC_50_ value ranging from 2.33–51.80 μM. In descending pattern, conjugates 5d, 5e and 5c were the most active conjugates against MCF-7 cells with IC_50_ values of 2.33, 3.03 and 7.39 μM, respectively. Interestingly, conjugates 5d and 5e showed comparable cytotoxic action with that of Dox against MCF-7 cells (IC_50_ = 6.89 μM). The activity of conjugates 5d and 5e was 2.96- and 2.27-fold that of Dox, respectively. In addition, the cytotoxic activity value of conjugate 5c (IC_50_ = 7.39 μM) was nearly equivalent to that of the standard Dox. Considering aryl group at C3 position of acrylamide moiety, conjugates 5a and 5b which include 4-chlorophenyl and 4-bromophenyl showed a significant drop in cytotoxic activity against MCF-7 *Br Ca* cell line, with IC_50_ value of 51.80 and 30.71 μM, respectively, in comparison to Dox (IC_50_ = 6.89 μM). In addition, the substituting 3-(4-bromophenyl)acrylamide for 3-(3,5-dibromo-4-hydroxyphenyl)acrylamide substituent in compound 5g (IC_50_ = 18.46 μM) results in a rise of the biological activity. Additionally, activity increased with the incorporation of a 4-nitrophenyl moiety at C3 position of acrylamide moiety; 5c (IC_50_ = 7.39 μM). On the other hand, the biological activity increased and became stronger with the aryl group having electron-releasing substituents at C3 position of acrylamide moiety in naphthalen-1-yloxyacetamide-3-(4-methoxyphenyl)acrylamide 5d (IC_50_ = 2.33 μM) and naphthalen-1-yloxyacetamide-3-(4-dimethylaminophenyl)acrylamide 5e (IC_50_ = 3.03 μM). Furthermore, the activity against MCF-7 *Br Ca* cells was lowered by 5h (IC_50_ = 25.90 μM) with 2,3,4-trimethoxyphenyl or its positional isomer 5i (IC_50_ = 16.94 μM) with 3,4,5-trimethoxyphenyl moiety. Again, conjugate 6a (IC_50_ = 9.18 μM) having furan-2-yl moiety at C3 position of acrylamide series had one third of Dox's cytotoxic action. Alternatively, with respect to arylamide moiety attached to C2 position of acrylamide moiety, the presence of 4-methoxybenzamide group in conjugates 5j (IC_50_ = 21.21 μM) and 6b (IC_50_ = 11.52 μM) decreased the activity compared to their corresponding 2-(unsubstituted benzamide) acrylamide in conjugates 5i (IC_50_ = 16.94 μM) and 6a (IC_50_ = 9.18 μM).

**Table 1:** The cytotoxicity screening results of investigated naphthalen-1-yloxyacetamide tethered 2,3,-disubstituted acrylamide conjugates 5a–j and 6a, 6b. Values represent the mean value ± SD (*n* = 3)

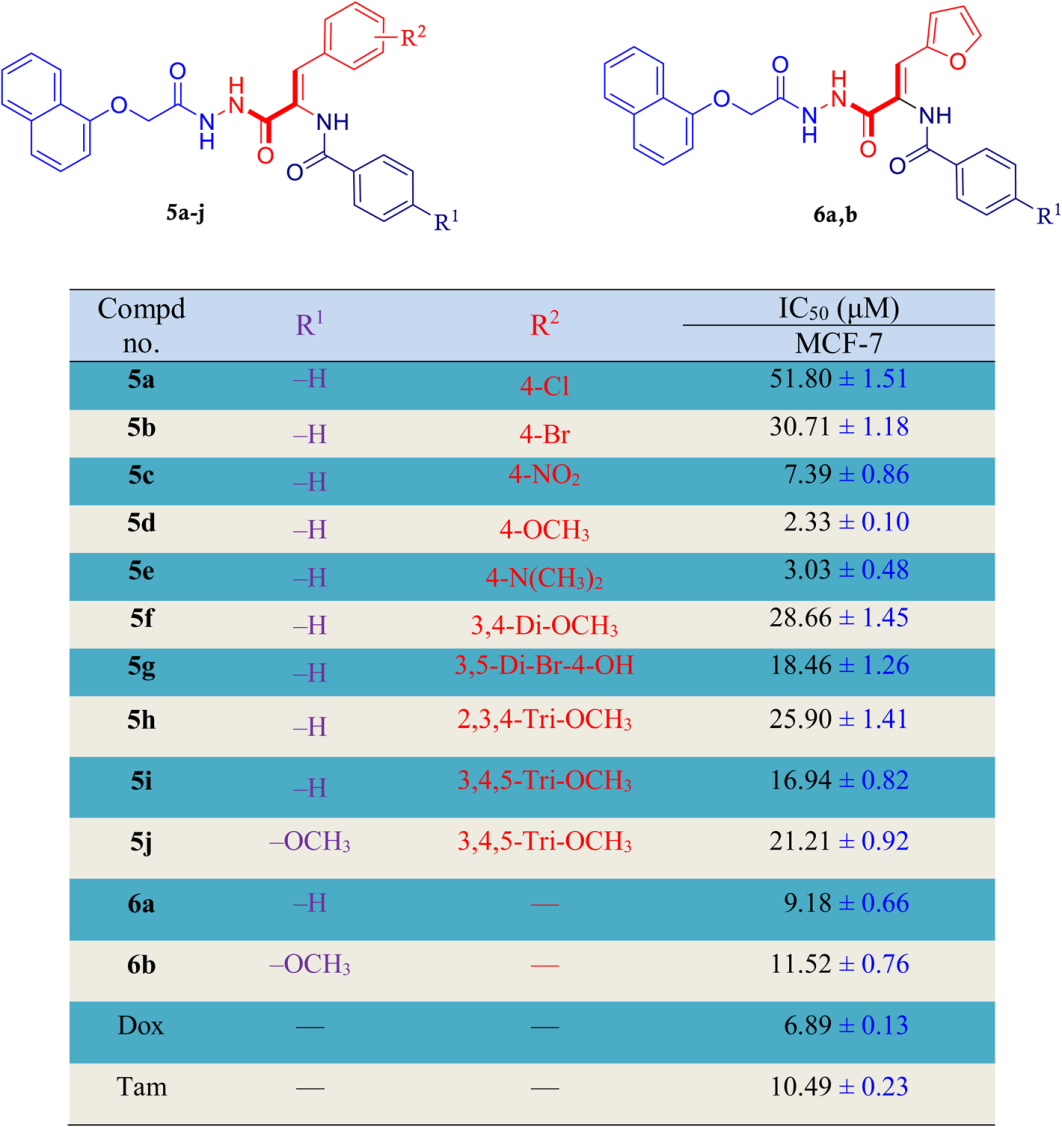

#### 2.2.2. Aromatase inhibition assay

**Fig. 3:**
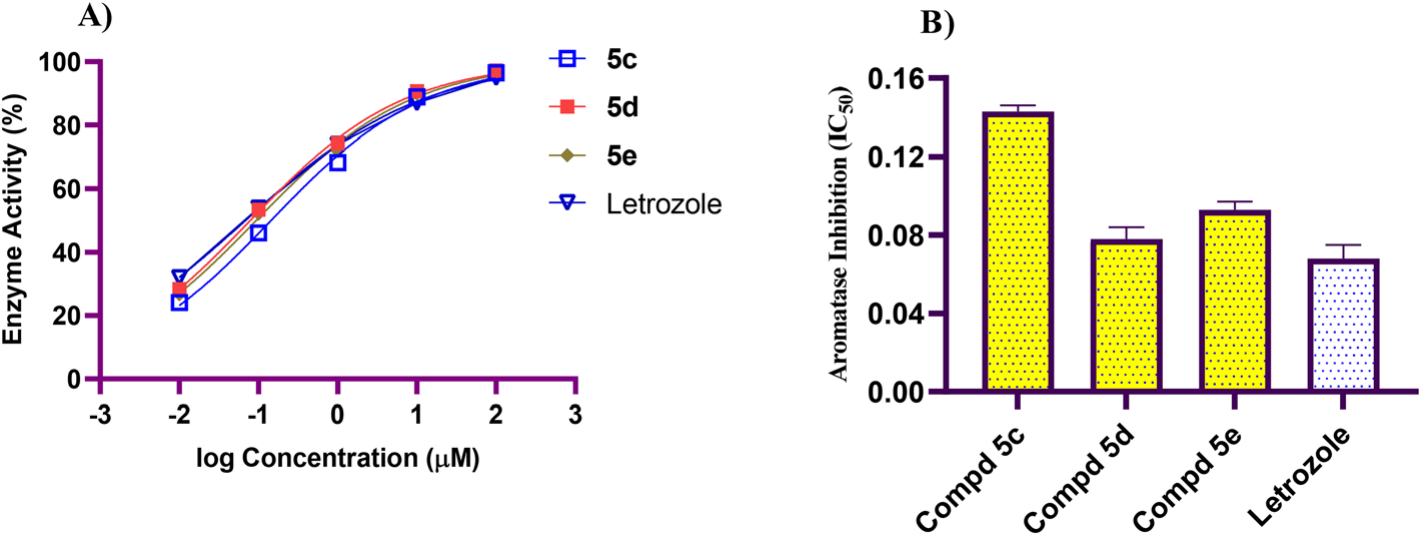
(A) The impact of naphthalen-1-yloxtacetamide-tethered acrylamides 5c, 5d, 5e and Letrozole at five different concentrations for IC_50_ determination of aromatase enzyme. (B) Graphical illustration of the IC_50_ values against aromatase enzyme in MCF-7 *Br Ca* cells treated with naphthalen-1-yloxtacetamide-tethered acrylamides 5c, 5d, and 5e compared with Letrozole.

Aromatase inhibitors are a potential path in searching for new therapies that can treat cancer, particularly hormone-dependent *Br Ca*.^41^ It has been shown that *Br Ca* cells displayed an enhanced expression of aromatase, and, thus, produce higher concentrations of estrogens than normal cells.^42^ Therefore, aromatase inhibitors are the first-line therapy for *Br Ca* management.^43^ In this regard, to determine whether constructed naphthalen-1-yloxyacetamide-tethered acrylamide conjugates trigger aromatase enzyme inhibition in MCF-7 *Br Ca* cells to induce apoptotic cellular death. The most potent conjugates 5c, 5d and 5e (at five different concentrations) were examined for their potency as aromatase inhibitors using ELISA method kit. Letrozole was utilized as reference standard in this study. Results presented in Fig. 3 clearly indicated the noteworthy decrease in the aromatase enzyme after treatment with investigated compounds 5c, 5d and 5e with IC_50_ ranges of 0.078–0.143 μM, when compared to the reference Letrozole (aromatase IC_50_ = 0.068 μM). Compound 5d the most potent antiproliferative agents, displayed the utmost inhibitory efficacy against aromatase with IC_50_ value of 0.078 μM, which was comparable to Letrozole (aromatase IC_50_ = 0.068 μM). The results also showed that, compared to Letrozole, the other investigated naphthalen-1-yloxytacetamide tethered acrylamides 5c and 5e showed good aromatase inhibition activity (IC_50_ values of 0.143 and 0.093 μM, respectively). The results of this assay method indicated that naphthalene-1-yloxtacetamide-acrylamides 5d and 5e had substantial antiproliferative activity against MCF-7 *Br Ca* cells and as aromatase inhibitors.

#### 2.2.3. *In vitro* DNA flow cytometry for compound **5d** on MCF-7 *Br Ca* cells

**Fig. 4:**
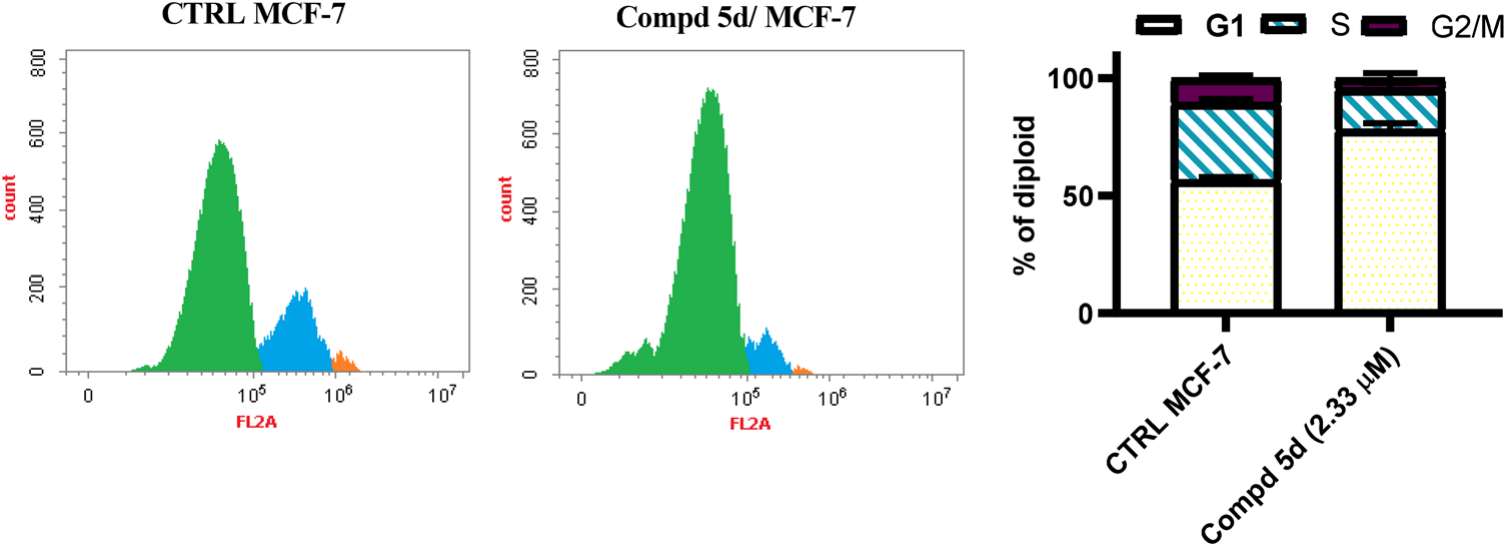
Cell cycle disruption after naphthalene-1-yloxyacetamide tethered 2-(benzamide)-3-(4-methoxypheny)acrylamide conjugate 5d treatment at indicated concentration analysis of MCF-7 *Br Ca* cell line.

It is frequently utilized in cell biology to comprehend growth regulation, cell proliferation and the impact of both drugs and genetic alterations on cellular division.^44^ Recognizing and manipulating the phase at which cell cycle arrest occurs can improve the therapeutic efficacy and selectivity of chemotherapeutic drugs, as many cancer therapies target rapidly proliferating cells.^45^ The most cytotoxic naphthalen-1-yloxyacetamide-tethered acrylamide conjugate, 5d, was chosen in order to further assess its impact on the advancement on cell cycle in MCF-7 *Br Ca* cells. In this context, MCF-7 *Br Ca* cells were hatched with the examined conjugate, 5d, at the IC_50_ value for 48 h, and then the DNA content was measured by flow cytometry utilizing FACS Calibur device. There was also a control experiment that receives no treatment. According to Flow cytometric study, naphthalen-1-yloxyacetamide-tethered acrylamide conjugate 5d treatment causes a G1 stoppage that is almost 1.4-times greater than that of the control (Fig. 4). The accumulation of cells in G1 phase suggest that conjugate 5d may play a part in inhibiting cellular proliferation and causing cell death. These findings showed that the analyzed naphthalen-1-yloxyacetamide-tethered acrylamide conjugate 5d inhibited cell proliferation at the G1 phase of the cellular cycle.

#### 2.2.4. *In vitro* apoptosis identification by FITC-Annexin V staining

**Fig. 5:**
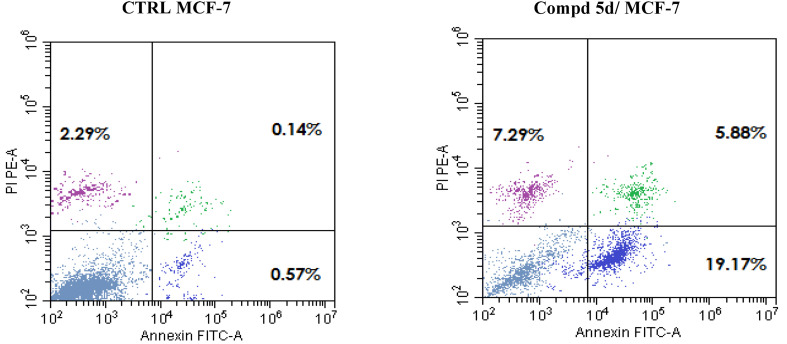
Cytofluorimetric analysis of MCF-7 *Br Ca* cell line treated with naphthalene-1-yloxyacetamide tethered 2-(benzamide)-3-(4-methoxypheny)acrylamide conjugate 5d at the IC_50_ concentration and compared to the negative controls.

Tissue homeostasis, immunological response, and appropriate development all depend on apoptosis.^46^ Chromosomal DNA fragmentation into tiny units known as oligomers, apoptotic body formation and nuclei condensation and fragmentation are all signs of apoptotic cell death.^47^ The abnormal suppression of apoptosis is a hallmark of cancer and autoimmune disease.^48^ One key strategy for cancer prevention or treatment has been to induce apoptosis either directly, or after final differentiation, or after stopping the cell cycle.^49^ To further evaluate the tumor suppression ability of naphthalen-1-yloxyacetamide tethered acrylamide conjugate 5d, and to verify that the found G1 cells accumulation was caused by apoptosis, *Br Ca* cells treated with 5d for 48 h was analyzed with Propidium Iodide (PI) and fluorochrome Annexin V stain for apoptosis determination. MCF-7 *Br Ca* cells treated with naphthalen-1-yloxyacetamide tethered acrylamide conjugate 5d revealed an increased activity of both early apoptosis and late apoptosis sections (Fig. 5). Interestingly, the late apoptotic cells percentage showed a notable rise after treatment with compound 5d from 0.14% in control untreated to 5.88% (42.0-fold increase). In addition, early apoptosis significantly boosted from 0.57% in the no treatment control to 19.17% (33.6-fold increase) upon treatment with conjugate 5d. The experiment's outcome was consistent with the previous one, confirming that naphthalen-1-yloxyacetamide-tethered acrylamide conjugate 5d demonstrated its cytotoxic impact *via* apoptosis-promoting activity.

#### 2.2.5. *In vitro* Bax and Bcl2 levels assay

**Fig. 6:**
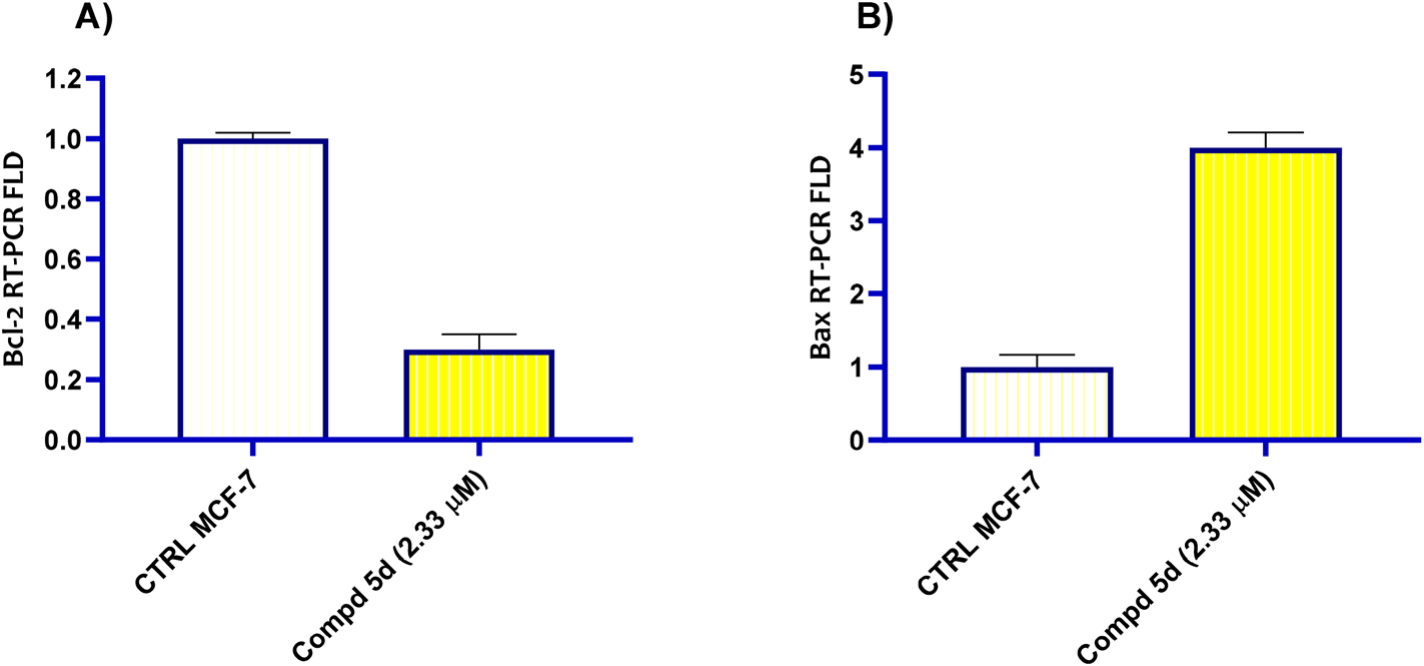
Bar graph of naphthalene-1-yloxyacetamide tethered 2-(benzamide)-3-(4-methoxypheny)acrylamide conjugate 5d treatment at indicated concentration analysis of Bcl-2 and Bax levels in MCF-7 *Br Ca* cell line. (A) Effect on Bcl2; (B) effect on Bax.

Programmed cell death, or apoptosis, is a complex series of biochemical processes.^50^ Pro-apoptotic proteins include Bad, Bax, Bid and Bim, while anti-apoptotic members include Bcl-2 and Bc-LXI.^51,52^ The anti-apoptotic proteins prevent cytochrome-c from release, which inhibits apoptosis, while pro-apoptotic members activate the release of cytochrome-*c*.^53^ When there is a higher ratio of pro-apoptotic proteins than anti-apoptotic ones, the outer mitochondrial membrane becomes permeable leading to a cascade of events.^54^ The cytochrome-*c* is released, which activates caspase 9, which in turn triggers caspase 3, which triggers apoptotic processes.^55^ In this context, the most active naphthalen-1-yloxyacetamide tethered acrylamide conjugate 5d was further studied for its impact on Bax and Bcl2 levels against MCF-7 *Br Ca* cell line using qRT-PCR technique. According to the data, the tested compound's Bax gen level increased noticeably compared with control. Compound 5d which was bearing 3-(4-methoxyphenyl)acrylamide moiety possessed an overexpression of Bax level with 4.0-fold higher than untreated MCF-7 *Br Ca* cells. However, in contrast to negative control group, conjugate 5d diminished the amount of Bcl-2 gene level in MCF-7 *Br Ca* cells by a factor of 0.3 (Fig. 6).

#### 2.2.6. Apoptotic markers activation

**Fig. 7:**
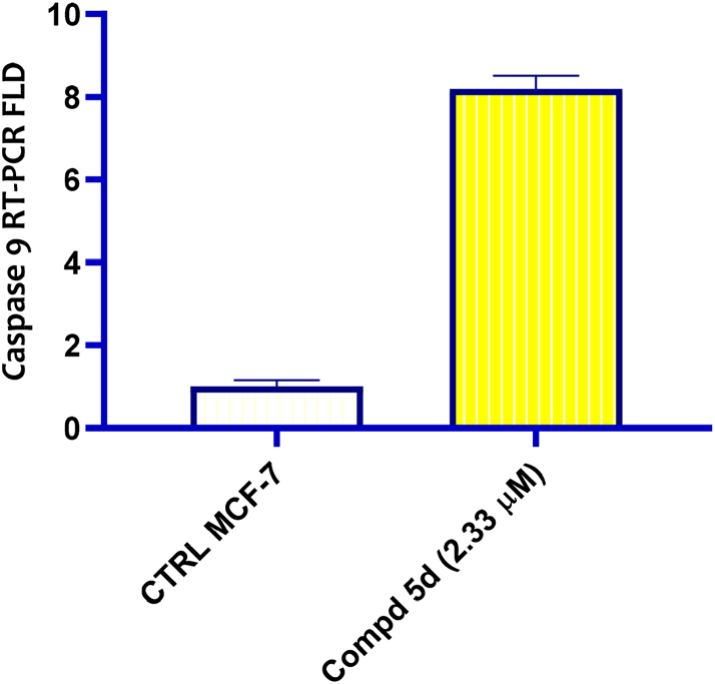
Bar graph of naphthalen-1-yloxyacetamide tethered 2-(benzamide)-3-(4-methoxypheny)acrylamide conjugate 5d treatment at indicated concentration analysis of caspase 9 level in MCF-7 *Br Ca* cell line using qRT-PCR technique.

An essential initiator caspase in the intrinsic apoptotic process is caspase 9.^56^ It is essential for inducing cell death in response to internal stress such as oxidative stress, DNA damage, or oncogene activation conditions, which are frequently present in cancer cells.^57^ Therefore, therapeutic strategies that enhance or restore its activity can potentially overcome apoptosis resistance in tumors.^58^ In this context, naphthalene-1-yloxyacetamide tethered acrylamide conjugate 5d were evaluated as caspase 9 activator against MCF-7 cells using qRT-PCR technique. The results are presented in Fig. 7. The results demonstrated that conjugate 5d which was bearing 3-(4-methoxyphenyl)acrylamide moiety attached naphthalene-1-yloxyacetamide function boosted caspase 9 gene level compared to the controls. It is worth mentioning that, the level of caspase 9 protein level is increased 8.2-fold more than the negative control. The aforementioned findings could be interpreted as evidence that the apoptosis may be attributed to the up-regulation of caspase 9 which induced by the tested naphthalen-1-yloxyacetamide tethered acrylamide conjugate 5d.

#### 2.2.7. *In silico* ADME prediction

**Fig. 8:**
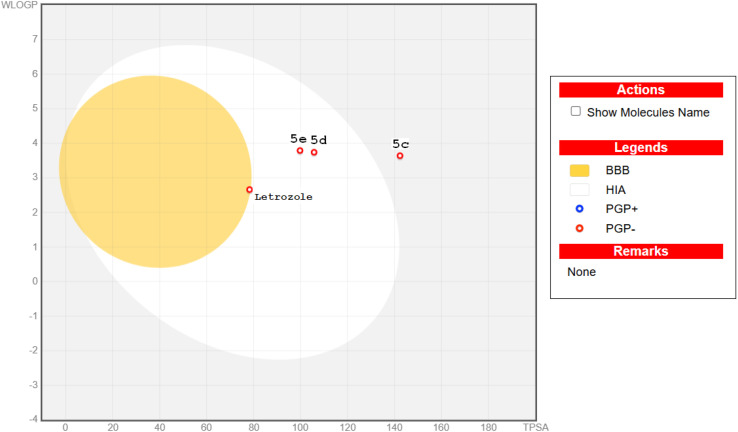
*In silico* ADME predictive plot of the synthesized naphthalen-1-yloxyacetamide-tethered acrylamides 5c, 5d, 5e and Letrozole from Swiss ADME online tool.

Using the Swiss ADME online tool created by the Swiss institute of bioinformatics (SIB), computer-assisted ADME profile and drug-like characteristic of the most potent conjugates 5c, 5d and 5e was carried out to evaluate the pharmacokinetic features. Letrozole was utilized as reference molecule. It was anticipated that naphthalen-1-yloxyacetamide-tethered acrylamides 5d and 5e to have high GIT absorption as it was positioned in the white region of human intestinal absorption (HIA) in boiled egg chart (Fig. 7). In addition, conjugates 5d and 5e demonstrated good predicted absorption levels, while compound 5c displayed poor range. Furthermore, the investigated conjugates 5c, 5d and 5e may not have the capacity to penetrate the BBB as it was located outside the yellow region which denotes the BBB penetration, and therefore, it might be used wisely for peripheral tumors with no CNS problems. Moreover, the red circle in Fig. 8 denoted that the investigated naphthalen-1-yloxyacetamide-tethered acrylamides 5c, 5d and 5e was not a substrate for P-glycoprotein (PGP), so it can't be pumped by it out of the cells into the gut lumen, into the bile, or into the urine. This prediction suggested that all the investigated conjugates may be expected to enhance intracellular drug accumulation and boost therapeutic efficacy. In conclusion, it was found that naphthalen-1-yloxyacetamide-tethered acrylamides 5d and 5e exhibited not only significant biological activity, but also an acceptable predicted ADME and physicochemical aspects.

## 3. Conclusions

In summary, a new set of naphthalen-1-yloxyacetamides 5a–j and 6a, 6b having a 2,3-disubstituted acrylamide moiety has been successfully synthesized by a facile method. These chemical conjugates were characterized by elemental analysis as well as ^1^H-NMR, ^13^C-NMR spectroscopic tools. In addition, these conjugates were evaluated for their antiproliferative activity against the MCF-7 *Br Ca* cell line using the MTT colorimetric technique. Among all the examined conjugates, 5c, 5d, and 5e demonstrated notable cytotoxic activity with IC_50_ values of 2.33, 3.03, and 7.39 μM, respectively, compared to the standard Dox (IC_50_ = 6.89 μM). The *in vitro* enzyme inhibitory assay of 5c, 5d, and 5e against aromatase inhibition was evaluated, and the obtained results were promising (IC_50_ values = 0.143, 0.078 and 0.093 μM, respectively) compared with Letrozole (IC_50_ = 0.068 μM) as standard aromatase inhibitory drug. Further, a representative promising conjugate in cytotoxic and aromatase inhibition assays was subjected to further mechanistic evaluation studies; cell cycle analysis was carried out on MCF-7 *Br Ca* cell line, which elicited G1 phase arrest. The mechanistic studies also demonstrated that naphthalen-1-yloxyacetamide 5d bearing a 2-benzamido-3-(4-methoxyphenyl)acrylamide moiety provoked apoptosis in MCF-7 *Br Ca* cells *via* the expressive downregulation of the anti-apoptotic protein Bcl-2, as well as increasing Bax and caspase 9 levels with regard to untreated controls. In conclusion, we identified 5d, which was bearing a 3-(4-methoxyphenyl)acrylamide moiety connected to naphthalen-1-yloxyacetamide function as a cytotoxic agent which inhibited aromatase enzyme and stopped the cell cycle at the point of G1. Hence, we may conclude that naphthalen-1-yloxyacetamide 5d, which contains a 2-benzamido-3-(4-methoxyphenyl)acrylamide moiety, was emerged as potential candidate for future *in vivo* investigation for pharmacokinetics and toxicity properties.

## 4. Experimental

### 4.1. Chemistry

#### 4.1.1. General reaction procedure for the synthesis of (*Z*)-*N*-(1-aryl-3-(2-(2-(naphthalen-1-yloxy)acetyl)hydrazineyl)-3-oxoprop-1-en-2-yl)arylamides 5a–j and 6a, 6b

To a suspension of naphthalen-1-yloxyacetohydrazide 3 (1.08 g, 5 mmol), respective ethyl 2-benzamido-3-(4-chlorophenyl)acrylate (1.65 g, 5 mmol) in pure ethanol (20 mL) was added catalytic amount of fused sodium acetate. The reaction mixture was refluxed until complete conversion as monitored by TLC (around 12–14 h). After cooled, the precipitates obtained were filtered, washed and crystallized with ethanol/H_2_O (3 : 1) to attain pure (*Z*)-*N*-(1-(4-chlorophenyl)-3-(2-(2-(naphthalen-1-yloxy)acetyl)hydrazineyl)-3-oxoprop-1-en-2-yl)benzamide (5a) as yellow solids. All the other naphthalene-1-yloxyacetamide-tethered acrylamide conjugates 5b–j and 6a, 6b were obtained in a similar reaction procedure of 5a in moderate to good yields.

##### 4.1.1.1. (*Z*)-*N*-(1-(4-Chlorophenyl)-3-(2-(2-(naphthalen-1-yloxy)acetyl)hydrazineyl)-3-oxoprop-1-en-2-yl)benzamide (5a)

Yellow solid, yield 77%; mp: 239–241 °C. ^1^H-NMR (400 MHz, DMSO-*d*_6_) *δ* 10.38 (s, 2H, 2NH), 10.01 (s, 1H, NH), 8.38 (dd, *J* = 15.9, 7.6 Hz, 1H), 8.01 (d, *J* = 7.4 Hz, 1H), 7.89 (d, *J* = 7.2 Hz, 2H), 7.61 (d, *J* = 8.0 Hz, 2H), 7.59–7.48 (m, 6H), 7.44 (t, *J* = 7.3 Hz, 3H), 7.28 (s, 1H, H_C=C_), 7.00 (d, *J* = 6.8 Hz, 1H), 4.85 (d, *J* = 9.8 Hz, 2H, O**CH**_2_N). MS (*m*/*z*, %): 499.48 (M^+^, 19.09), 186.08 (100). Analysis: calc. for C_28_H_22_ClN_3_O_4_ (499.95): C 67.27, H 4.44, N 8.41%, found: C 67.41, H 4.52, N 8.32%.

##### 4.1.1.2. (*Z*)-*N*-(1-(4-Bromophenyl)-3-(2-(2-(naphthalen-1-yloxy)acetyl)hydrazineyl)-3-oxoprop-1-en-2-yl)benzamide (5b)

Orange solid, yield 82%; mp: 222–224 °C. ^1^H-NMR (400 MHz, DMSO-*d*_6_) *δ* 10.35 (s, 2H, 2NH), 10.02 (s, 1H, NH), 8.39–8.33 (m, 1H), 8.02–7.99 (m, 1H), 7.93–7.84 (m, 2H), 7.63–7.59 (m, 2H), 7.57 (s, 2H), 7.54 (d, *J* = 2.9 Hz, 3H), 7.53–7.51 (m, 2H), 7.42 (td, *J* = 7.9, 3.9 Hz, 2H), 7.25 (s, 1H, H_C=C_), 6.99 (dd, *J* = 7.7, 4.6 Hz, 1H), 4.85 (d, *J* = 8.5 Hz, 2H, O**CH**_2_N). ^13^C-NMR (101 MHz, DMSO-*d*_6_) *δ*: 167.06, 166.35, 164.68, 153.82, 134.49, 133.98, 133.80, 132.23, 131.99, 131.75, 129.94, 129.19, 128.76, 128.42, 127.83, 127.01, 126.47, 125.77, 125.29, 122.59, 122.51, 121.16, 106.15, 66.85. MS (*m*/*z*, %): 544.94 (M^+^, 20.69), 441.08 (100). Analysis: calc. for C_28_H_22_BrN_3_O_4_ (544.41): C 61.78, H 4.07, N 7.72%, found: C 61.65, H 3.96, N 7.86%.

##### 4.1.1.3. (*Z*)-*N*-(3-(2-(2-(Naphthalen-1-yloxy)acetyl)hydrazineyl)-1-(4-nitrophenyl)-3-oxoprop-1-en-2-yl)benzamide (5c)

Pale yellow solid, yield 85%; mp: 247–249 °C. ^1^H-NMR (400 MHz, DMSO-*d*_6_) *δ* 10.44 (s, 2H, 2NH), 10.19 (s, 1H, NH), 8.38–8.33 (m, 1H), 8.25–8.21 (m, 2H), 8.02–7.97 (m, 2H), 7.91–7.88 (m, 1H), 7.83 (d, *J* = 8.8 Hz, 2H), 7.61 (t, *J* = 7.4 Hz, 1H), 7.57–7.54 (m, 3H), 7.52 (d, *J* = 4.3 Hz, 2H), 7.42 (t, *J* = 8.1 Hz, 1H), 7.30 (s, 1H, H_C=C_), 7.02–6.96 (m, 1H), 4.85 (s, 2H, O**CH**_2_N). ^13^C-NMR (101 MHz, DMSO-*d*_6_) *δ*: 167.14, 166.42, 164.45, 153.82, 147.17, 141.56, 134.50, 133.79, 132.53, 132.38, 130.73, 128.80, 128.48, 127.84, 127.27, 127.03, 126.48, 125.78, 125.30, 124.12, 122.50, 121.17, 106.15, 66.83. MS (*m*/*z*, %): 510.91 (M^+^, 38.18), 80.00 (100). Analysis: calc. for C_28_H_22_N_4_O_6_ (510.51): C 65.88, H 4.34, N 10.97%, found: C 66.02, H 4.48, N 10.84%.

##### 4.1.1.4. (*Z*)-*N*-(1-(4-methoxyphenyl)-3-(2-(2-(naphthalen-1-yloxy)acetyl)hydrazineyl)-3-oxoprop-1-en-2-yl)benzamide (5d)

Orange solid, yield 79%; mp: 224–226 °C. ^1^H-NMR (400 MHz, DMSO-*d*_6_) *δ* 10.33 (s, 1H, NH), 10.14 (s, 1H, NH), 9.89 (s, 1H, NH), 8.44–8.37 (m, 1H), 8.03 (d, *J* = 7.3 Hz, 2H), 7.90 (dd, *J* = 6.7, 2.6 Hz, 2H), 7.59–7.56 (m, 2H), 7.55–7.51 (m, 4H), 7.43 (t, *J* = 8.0 Hz, 2H), 7.32 (s, 1H, H_C=C_), 6.97 (dd, *J* = 21.6, 8.0 Hz, 3H), 4.86 (s, 2H, O**CH**_2_N), 3.76 (s, 3H, OCH_3_). ^13^C-NMR (101 MHz, DMSO-*d*_6_) *δ*: 167.11, 166.27, 164.96, 160.19, 153.73, 134.51, 132.02, 131.69, 130.85, 128.70, 128.41, 128.38, 127.83, 127.07, 127.04, 126.47, 125.78, 125.29, 125.27, 122.60, 121.26, 114.51, 106.21, 66.98, 55.68. MS (*m*/*z*, %): 495.40 (M^+^, 18.67), 69.11 (100). Analysis: calc. for C_29_H_25_N_3_O_5_ (495.54): C 70.29, H 5.09, N 8.48%, found: C 70.17, H 5.21, N 8.63%.

##### 4.1.1.5. (*Z*)-*N*-(1-(4-(Dimethylamino)phenyl)-3-(2-(2-(naphthalen-1-yloxy)acetyl)hydrazineyl)-3-oxoprop-1-en-2-yl)benzamide (5e)

Red solid, yield 80%; mp: 228–230 °C. ^1^H-NMR (400 MHz, DMSO-*d*_6_) *δ*: 11.36 (s, 1H, NH), 10.32 (s, 1H, NH), 8.23 (d, *J* = 8.7 Hz, 1H), 7.93 (dt, *J* = 7.1, 1.4 Hz, 2H), 7.89–7.85 (m, 2H), 7.78 (dd, *J* = 18.3, 8.2 Hz, 2H), 7.52–7.48 (m, 2H), 7.44–7.38 (m, 3H), 7.34–7.28 (m, 2H), 7.20 (s, 1H, H_C=C_), 6.83 (d, *J* = 8.9 Hz, 2H), 4.91 (d, *J* = 7.8 Hz, 2H, O**CH**_2_N), 3.06 (s, 6H, N(**CH**_3_)_2_). ^13^C-NMR (101 MHz, DMSO-*d*_6_) *δ*: 168.02, 167.92, 167.25, 156.43, 155.75, 152.41, 135.32, 134.42, 131.76, 130.92, 129.80, 129.14, 128.24, 128.00, 127.34, 127.25, 126.95, 124.38, 121.73, 119.04, 112.30, 108.04, 107.82, 66.61, 40.07. MS (*m*/*z*, %): 508.92 (M^+^, 10.34), 105.11 (100). Analysis: calc. for C_30_H_28_N_4_O_4_ (508.58): C 70.85, H 5.55, N 11.02%, found: C 70.97, H 5.39, N 10.88%.

##### 4.1.1.6. (*Z*)-*N*-(1-(3,4-Dimethoxyphenyl)-3-(2-(2-(naphthalen-1-yloxy)acetyl)hydrazineyl)-3-oxoprop-1-en-2-yl)benzamide (5f)

Orange solid, yield 74%; mp: 217–219 °C. ^1^H-NMR (400 MHz, DMSO-*d*_6_) *δ*: 10.74 (s, 1H, NH, NH), 10.38 (s, 1H, NH, NH), 9.86 (s, 1H, NH, NH), 8.44 (dd, *J* = 8.5, 1.4 Hz, 1H), 8.29–8.24 (m, 2H), 8.17–8.14 (m, 1H), 8.06 (dd, *J* = 8.8, 1.5 Hz, 2H), 7.86 (ddd, *J* = 8.4, 6.8, 1.4 Hz, 1H), 7.79 (d, *J* = 8.6 Hz, 1H), 7.75–7.67 (m, 1H), 7.62 (d, *J* = 8.9 Hz, 1H), 7.40 (s, 1H), 7.24 (s, 1H, H_C=C_), 7.17 (d, *J* = 8.9 Hz, 1H), 7.09 (t, *J* = 9.0 Hz, 2H), 6.96 (d, *J* = 8.9 Hz, 1H), 3.88 (s, 2H, OCH_2_, O**CH**_2_N), 3.85 (s, 3H, OCH_3_), 3.77 (s, 3H, OCH_3_). ^13^C-NMR (101 MHz, DMSO-*d*_6_) *δ*: 166.15, 165.95, 165.41, 163.88, 162.46, 162.11, 160.29, 155.00, 148.30, 142.11, 137.77, 134.75, 132.45, 131.81, 131.04, 130.41, 129.73, 127.95, 126.96, 124.22, 117.13, 115.37, 115.11, 114.57, 113.98, 56.17, 55.92, 55.71. MS (*m*/*z*, %): 525.05 (M^+^, 29.49), 225.40 (100). Analysis: calc. for C_30_H_27_N_3_O_6_ (525.56): C 68.56, H 5.18, N 8.00%, found: C 68.66, H 5.03, N 7.83%.

##### 4.1.1.7. (*Z*)-*N*-(1-(3,5-Dibromo-4-hydroxyphenyl)-3-(2-(2-(naphthalen-1-yloxy)acetyl)hydrazineyl)-3-oxoprop-1-en-2-yl)benzamide (5g)

Yellow solid, yield 73%; mp: 233–235 °C. ^1^H-NMR (400 MHz, DMSO-*d*_6_) *δ* 10.58 (s, 1H, NH), 10.40 (s, 1H, OH), 10.35 (s, 1H, NH), 10.11 (s, 1H, NH), 8.38 (dd, *J* = 15.8, 7.9 Hz, 1H), 7.99 (dd, *J* = 14.0, 5.8 Hz, 3H), 7.94–7.86 (m, 2H), 7.82 (s, 1H), 7.59 (d, *J* = 12.6 Hz, 2H), 7.53 (d, *J* = 7.9 Hz, 3H), 7.45–7.40 (m, 1H), 7.27 (d, *J* = 13.1 Hz, 1H, H_C=C_), 6.99 (dd, *J* = 7.7, 5.1 Hz, 1H), 4.89–4.84 (m, 2H, O**CH**_2_N). ^13^C-NMR (101 MHz, DMSO-*d*_6_) *δ*: 167.66, 167.13, 167.04, 153.82, 153.73, 135.63, 134.50, 133.95, 133.73, 133.38, 131.46, 128.78, 128.32, 127.83, 127.02, 126.47, 125.78, 125.30, 122.52, 121.17, 117.59, 112.18, 106.14, 66.87. MS (*m*/*z*, %): 639.65 (M^+^, 10.70), 92.29 (100). Analysis: calc. for C_28_H_21_Br_2_N_3_O_5_ (639.30): C 52.61, H 3.31, N 6.57%, found: C 52.50, H 3.43, N 6.71%.

##### 4.1.1.8. (*Z*)-*N*-(3-(2-(2-(Naphthalen-1-yloxy)acetyl)hydrazineyl)-3-oxo-1-(2,3,4-trimethoxyphenyl)prop-1-en-2-yl)benzamide (5h)

Orange solid, yield 75%; mp: 211–213 °C. ^1^H-NMR (400 MHz, DMSO-*d*_6_) *δ* 10.27 (s, 2H, 2NH), 9.86 (s, 1H, NH), 8.40–8.35 (m, 1H), 8.00 (d, *J* = 7.7 Hz, 2H), 7.89 (dd, *J* = 6.7, 3.0 Hz, 1H), 7.56–7.52 (m, 5H), 7.43 (d, *J* = 3.0 Hz, 2H), 7.39–7.34 (m, 1H, H_C=C_), 7.00 (dd, *J* = 8.0, 3.2 Hz, 2H), 6.84–6.74 (m, 1H), 4.85 (d, *J* = 11.2 Hz, 2H, O**CH**_2_N), 3.85 (s, 3H, OCH_3_), 3.78 (s, 3H, OCH_3_), 3.76 (s, 3H, OCH_3_). ^13^C-NMR (101 MHz, DMSO-*d*_6_) *δ*: 167.10, 166.30, 164.89, 154.62, 153.83, 152.73, 150.13, 149.29, 142.06, 134.50, 132.06, 130.05, 128.68, 128.42, 127.88, 127.01, 126.51, 125.76, 125.29, 124.15, 122.60, 121.27, 121.14, 108.44, 106.21, 66.97, 61.88, 60.90, 56.38. MS (*m*/*z*, %): 555.93 (M^+^, 41.18), 85.64 (100). Analysis: calc. for C_31_H_29_N_3_O_7_ (555.59): C 67.02, H 5.26, N 7.56%, found: C 66.86, H 5.38, N 7.69%.

##### 4.1.1.9. (*Z*)-*N*-(3-(2-(2-(Naphthalen-1-yloxy)acetyl)hydrazineyl)-3-oxo-1-(3,4,5-trimethoxyphenyl)prop-1-en-2-yl)benzamide (5i)

Orange solid, yield 81%; mp: 216–218 °C. ^1^H-NMR (400 MHz, DMSO-*d*_6_) *δ*: 10.41 (s, 1H, NH), 10.26 (s, 1H, NH), 10.00 (s, 1H, NH), 8.42–8.33 (m, 1H), 8.08 (d, *J* = 7.7 Hz, 2H), 7.91–7.87 (m, 1H), 7.64–7.56 (m, 2H), 7.53 (d, *J* = 7.0 Hz, 4H), 7.42 (t, *J* = 8.0 Hz, 1H), 7.38 (s, 1H, H_C=C_), 7.02 (d, *J* = 6.6 Hz, 1H), 6.98 (s, 2H), 4.84 (s, 2H), 3.66 (s, 3H, OCH_3_), 3.61 (s, 6H, 2OCH_3_). ^13^C-NMR (101 MHz, DMSO-*d*_6_) *δ*: 166.84, 166.32, 164.52, 153.87, 153.04, 138.59, 134.50, 134.07, 132.20, 131.42, 129.80, 128.82, 128.67, 128.36, 127.83, 127.00, 126.48, 125.75, 125.31, 122.52, 121.11, 107.62, 106.16, 66.98, 60.51, 56.04. MS (*m*/*z*, %): 585.32 (M^+^, 33.92), 137.89 (100). Analysis: calc. for C_31_H_29_N_3_O_7_ (555.59): C 67.02, H 5.26, N 7.56%, found: C 67.14, H 5.33, N 7.46%.

##### 4.1.1.10. (*Z*)-4-Methoxy-*N*-(3-(2-(2-(naphthalen-1-yloxy)acetyl)hydrazineyl)-3-oxo-1-(3,4,5-trimethoxyphenyl)prop-1-en-2-yl)benzamide (5j)

Orange solid, yield 76%; mp: 225–227 °C. ^1^H-NMR (400 MHz, DMSO-*d*_6_) *δ*: 10.32 (s, 1H, NH), 10.08 (s, 1H, NH), 9.82 (s, 1H, NH), 8.36 (d, *J* = 7.8 Hz, 1H), 8.05 (d, *J* = 8.6 Hz, 2H), 7.91–7.87 (m, 1H), 7.59–7.53 (m, 2H), 7.52 (s, 1H), 7.42 (t, *J* = 7.9 Hz, 1H), 7.33 (s, 1H, H_C=C_), 7.05 (d, *J* = 8.7 Hz, 2H), 7.01 (d, *J* = 7.0 Hz, 1H), 6.97 (s, 2H), 4.83 (s, 2H, O**CH**_2_N), 3.84 (s, 3H, OCH_3_), 3.66 (s, 3H, OCH_3_), 3.61 (s, 6H, 2OCH_3_). ^13^C-NMR (101 MHz, DMSO-*d*_6_) *δ*: 166.89, 165.85, 164.73, 162.43, 153.85, 153.02, 138.53, 134.49, 130.31, 129.86, 127.83, 127.00, 126.48, 126.36, 125.75, 125.30, 122.52, 121.12, 114.06, 113.86, 107.77, 107.60, 106.16, 66.92, 60.51, 56.06, 55.89. MS (*m*/*z*, %): 585.59 (M^+^, 14.76), 93.44 (100). Analysis: calc. for C_32_H_31_N_3_O_8_ (585.61): C 65.63, H 5.34, N 7.18%, found: C 65.43, H 5.52, N 7.36%.

##### 4.1.1.11. (*Z*)-*N*-(1-(Furan-2-yl)-3-(2-(2-(naphthalen-1-yloxy)acetyl)hydrazineyl)-3-oxoprop-1-en-2-yl)benzamide (6a)

Pale yellow solid, yield 83%; mp: 234–236 °C. ^1^H-NMR (400 MHz, DMSO-*d*_6_) *δ*: 10.34 (s, 1H, NH), 10.24 (s, 1H, NH), 9.89 (s, 1H, NH), 8.38–8.33 (m, 1H), 8.08–8.04 (m, 2H), 7.90–7.87 (m, 1H), 7.78 (d, *J* = 1.8 Hz, 1H), 7.63–7.58 (m, 2H), 7.55 (d, *J* = 1.7 Hz, 1H), 7.54–7.53 (m, 2H), 7.52–7.51 (m, 1H), 7.43 (d, *J* = 8.0 Hz, 1H), 7.24 (s, 1H, H_C=C_), 6.99 (t, *J* = 6.6 Hz, 1H), 6.76 (d, *J* = 3.5 Hz, 1H), 6.60 (dd, *J* = 3.5, 1.8 Hz, 1H), 4.83 (s, 2H, O**CH**_2_N). ^13^C-NMR (101 MHz, DMSO-*d*_6_) *δ*: 167.06, 166.25, 164.21, 153.81, 150.00, 145.30, 134.49, 134.31, 132.09, 128.72, 128.43, 127.82, 127.01, 126.47, 126.22, 125.76, 125.29, 122.52, 121.15, 119.13, 114.96, 112.82, 106.14, 66.86. MS (*m*/*z*, %): 455.83 (M^+^, 36.14), 81.27 (100). Analysis: calc. for C_26_H_21_N_3_O_5_ (455.47): C 68.56, H 4.65, N 9.23%, found: C 68.74, H 4.51, N 9.38%.

##### 4.1.1.12. (*Z*)-*N*-(1-(Furan-2-yl)-3-(2-(2-(naphthalen-1-yloxy)acetyl)hydrazineyl)-3-oxoprop-1-en-2-yl)-4-methoxybenzamide (6b)

Pale yellow solid, yield 87%; mp: 242–244 °C. ^1^H-NMR (400 MHz, DMSO-*d*_6_) *δ*: 10.35 (s, 1H, NH, NH), 9.77 (s, 1H, NH, NH), 9.57 (s, 1H, NH, NH), 8.38 (dd, *J* = 11.6, 5.7 Hz, 1H), 8.12–8.01 (m, 2H), 7.89 (dd, *J* = 7.2, 3.5 Hz, 1H), 7.78 (d, *J* = 4.5 Hz, 1H), 7.55 (dt, *J* = 9.1, 4.1 Hz, 3H), 7.45–7.40 (m, 1H), 7.22 (d, *J* = 4.3 Hz, 1H, H_C=C_), 7.08 (d, *J* = 8.4 Hz, 2H), 7.00 (d, *J* = 6.9 Hz, 1H), 6.74 (t, *J* = 4.1 Hz, 1H), 6.65–6.55 (m, 1H), 4.94–4.81 (m, 2H, O**CH**_2_N), 3.86 (s, 3H, OCH_3_). ^13^C-NMR (101 MHz, DMSO-*d*_6_) *δ*: 167.07, 166.94, 165.67, 164.26, 162.42, 153.85, 150.13, 145.08, 134.50, 130.37, 127.83, 127.00, 126.47, 125.75, 125.30, 122.53, 121.13, 118.66, 114.58, 113.96, 112.80, 106.14, 105.94, 66.92, 55.89. MS (*m*/*z*, %): 485.82 (M^+^, 46.32), 319.23 (100). Analysis: calc. for C_27_H_23_N_3_O_6_ (485.50): C 66.80, H 4.78, N 8.66%, found: C 67.04, H 4.89, N 8.51%.

### 4.2. Biological activity

The biological evaluation of the constructed naphthalen-1-yloxyacetamide-tethered acrylamide derivatives 5a–j and 6a, 6b was applied as presented in the SI.

## Conflicts of interest

The authors have declared no conflict of interest.

## Supplementary Material

RA-015-D5RA06524K-s001

## Data Availability

The authors confirm that the data supporting the findings of this study are available within the article and/or its supporting information (SI). Supplementary information is available. See DOI: https://doi.org/10.1039/d5ra06524k.
