# Supplementary material for: Design, synthesis and anticancer evaluation of naphthalen-1-yloxyacetamide derivatives against MCF-7 cells
Source: RSC Adv. 2025 Sep 30;15(43):36152–64. doi: 10.1039/d5ra06524k (PMC12481249; doi:10.1039/d5ra06524k)
Supplement: RA-015-D5RA06524K-s001 [file RA-015-D5RA06524K-s001.pdf]

Supplementary Information (SI) for RSC Advances.
This journal is © The Royal Society of Chemistry 2025

Supporting Information

Design, Synthesis and Anticancer Evaluation of Naphthalen-1-yloxyacetamide Derivatives against MCF-7 Cells

Maha Ali Alghamdi [1], Mustafa R. Abdulbaqi [2], Rana Abdullah Alghamdi [3,4], Eman Fayad [1], Dalal Nasser Binjawhar [5], Hanadi A. Katouah [6], Abdullah Yahya Abdullah Alzahrani [7] and Amal M. Youssef Moustafa [8,*]

[1] Department of Biotechnology, College of Sciences, Taif University, P.O. Box 11099, Taif 21944, Saudi Arabia; [2] Department of Pharmaceutics, College of Pharmacy, Al-Naji University, Baghdad 10015, Iraq; [3] Department of Chemistry, Science and Arts College, King Abdulaziz University, Rabigh, Saudi Arabia; [4] Regenerative Medicine Unit, King Fahd Medical Research Centre, King Abdulaziz University, Jeddah, Saudi Arabia; [5] Department of Chemistry, College of science, Princess Nourah bint Abdulrahman University, P.O. Box 84428, Riyadh 11671, Saudi Arabia; [6] Chemistry Department, College of Science, Umm Al-Qura University, 21955, Makkah, Saudi Arabia; [7] Faculty of Science and Arts, Mohail Asser, King Khalid University, Saudi Arabia; [8] Chemistry Department, Faculty of Science, Port Said University, Port Said, Egypt.

** To whome correspondence should be addressed*

Amal M. Y. Moustafa, PhD. Chemistry Department, Faculty of Science, Port Said University, Port Said, Egypt.

E-mail address**:** amalyoussef840@gmail.com **(**Amal M. Y. Moustafa**)**

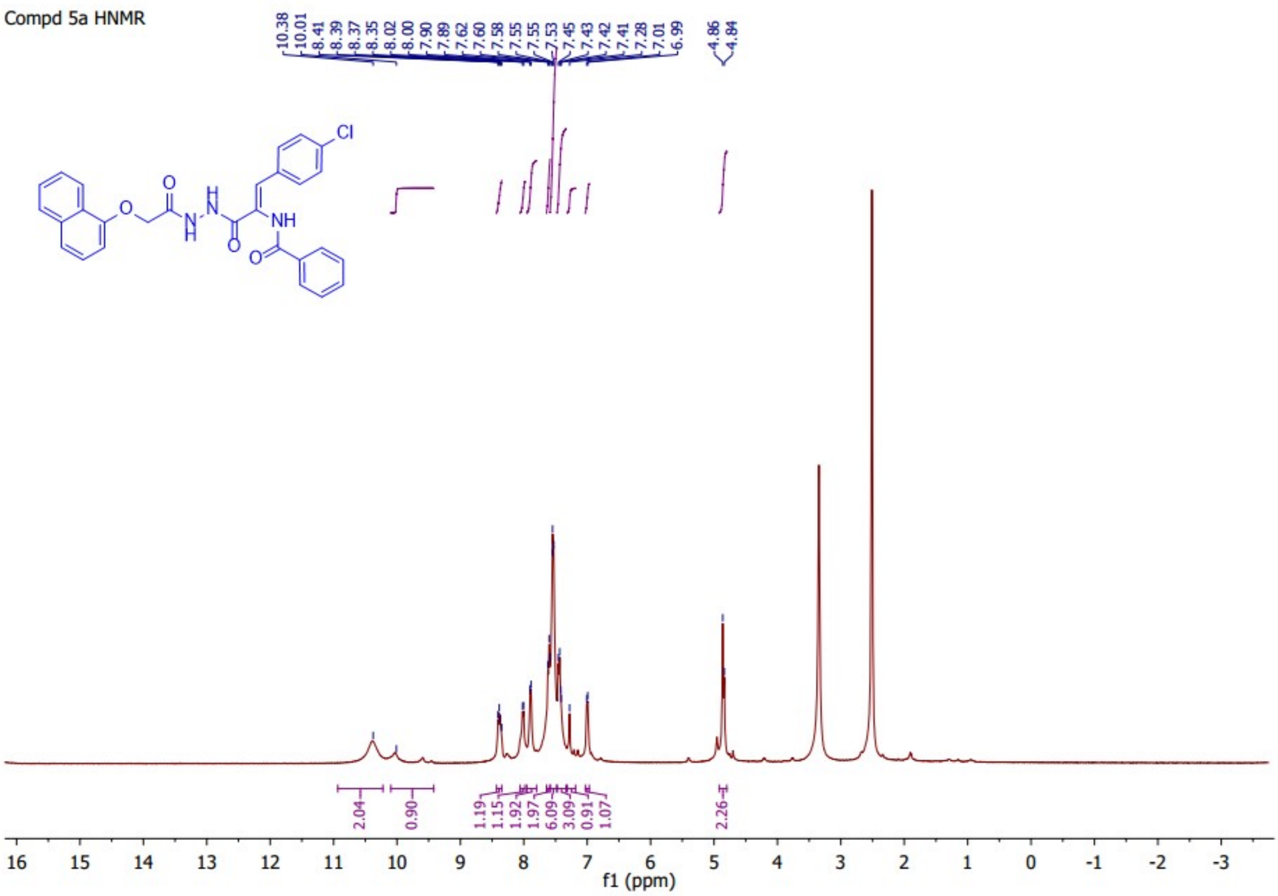

Figure S1: ^{1}H-NMR spectrum of compound **5a**

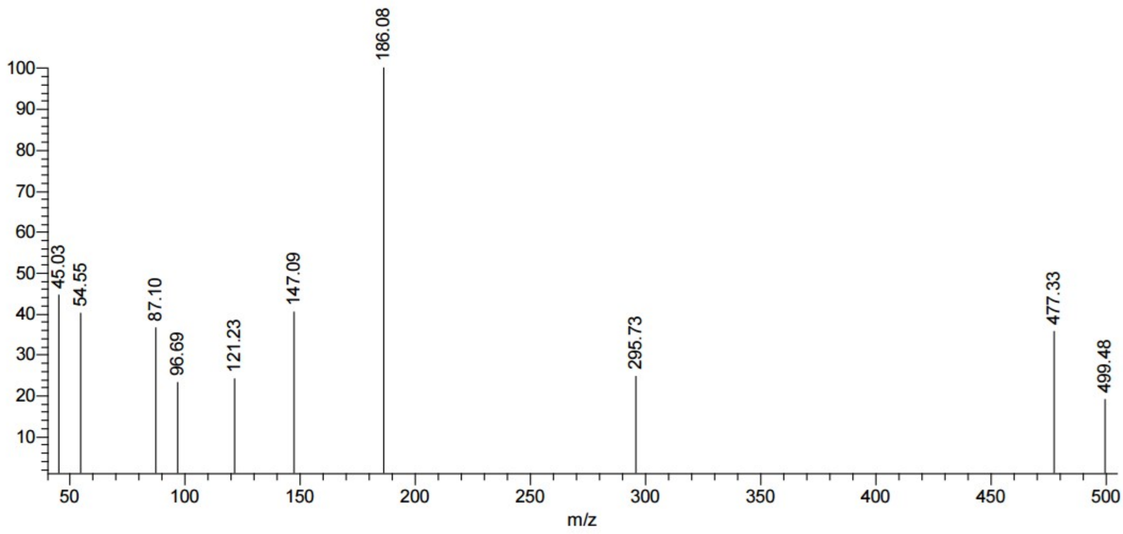

Figure S2: Mass spectrum of compound **5a**

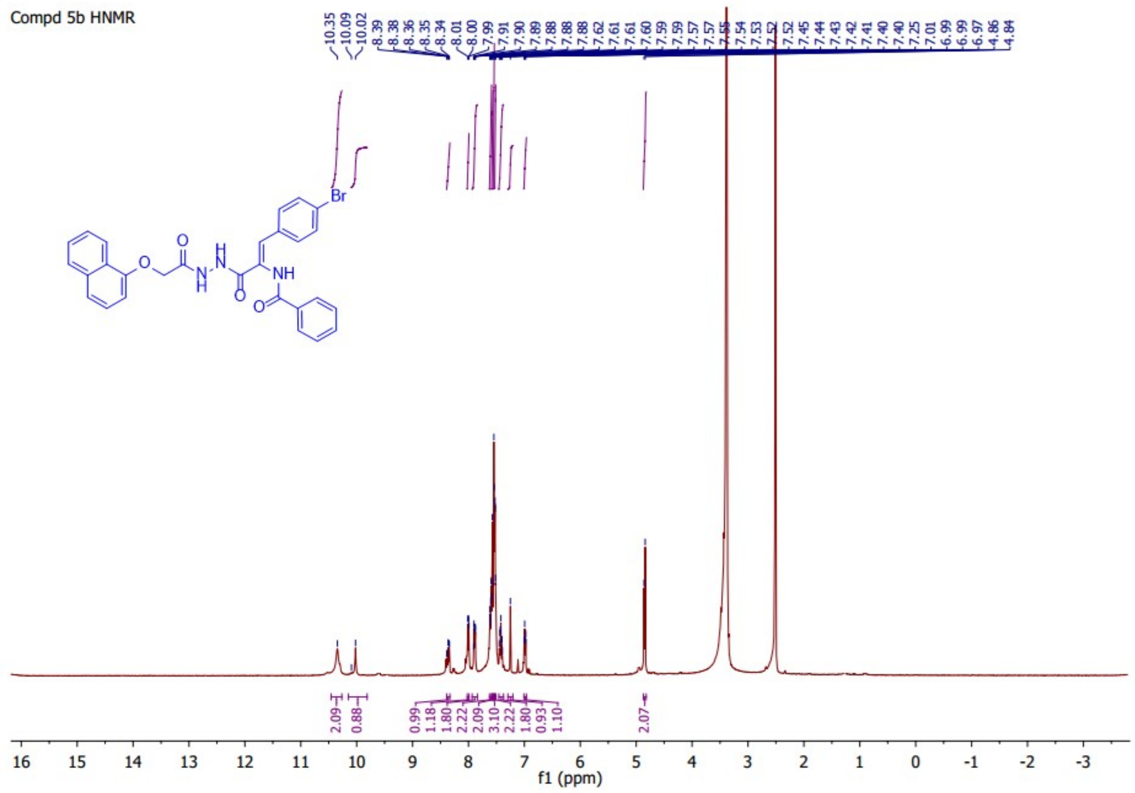

Figure S3: ^{1}H-NMR spectrum of compound **5b**

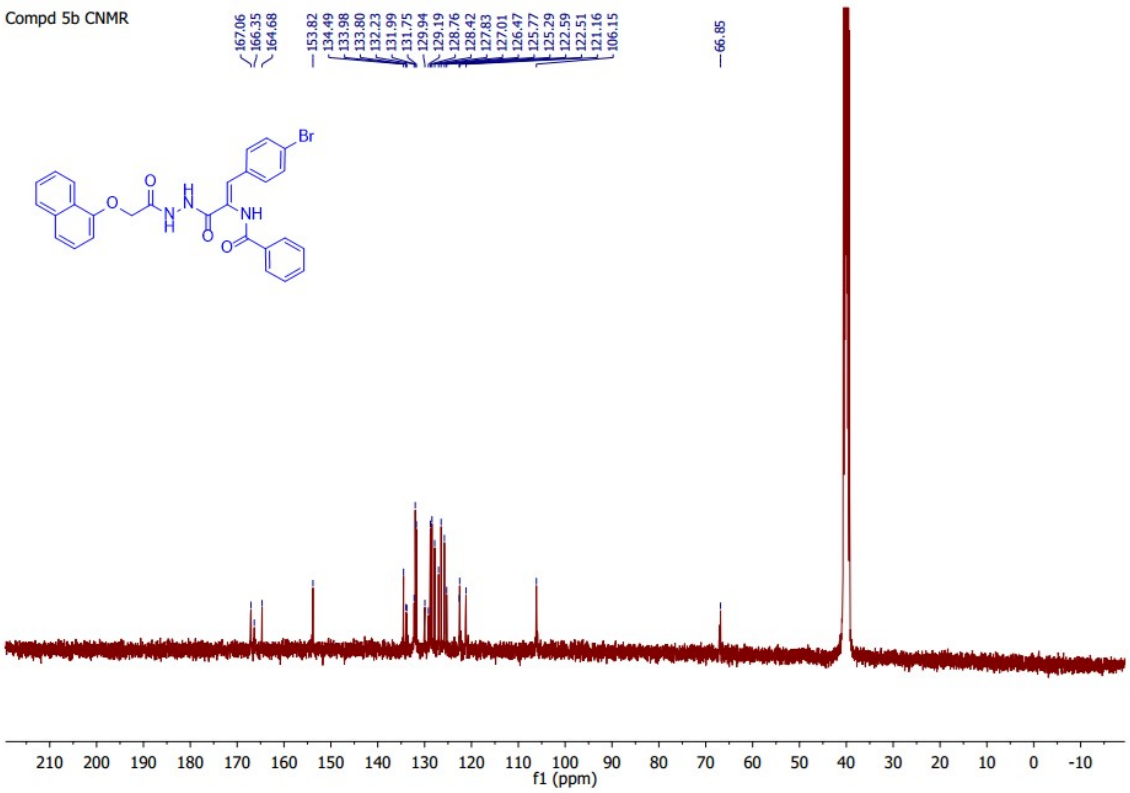

Figure S4: ^{13}C-NMR spectrum of compound **5b**

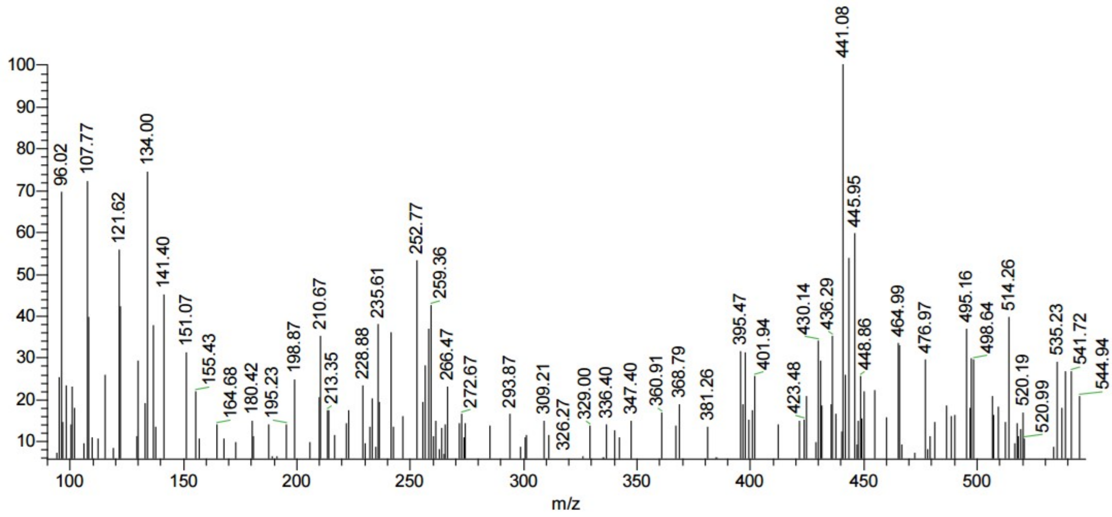

Figure S5: Mass spectrum of compound **5b**

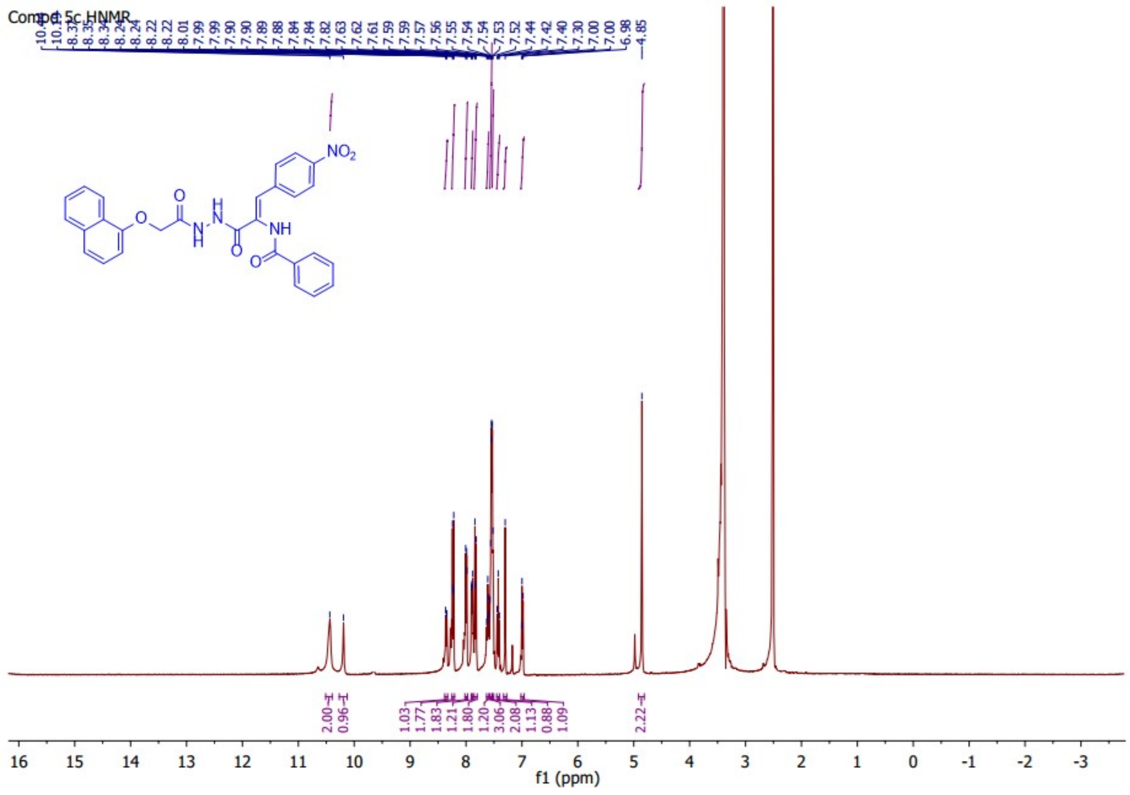

Figure S6: ^{1}H-NMR spectrum of compound **5c**

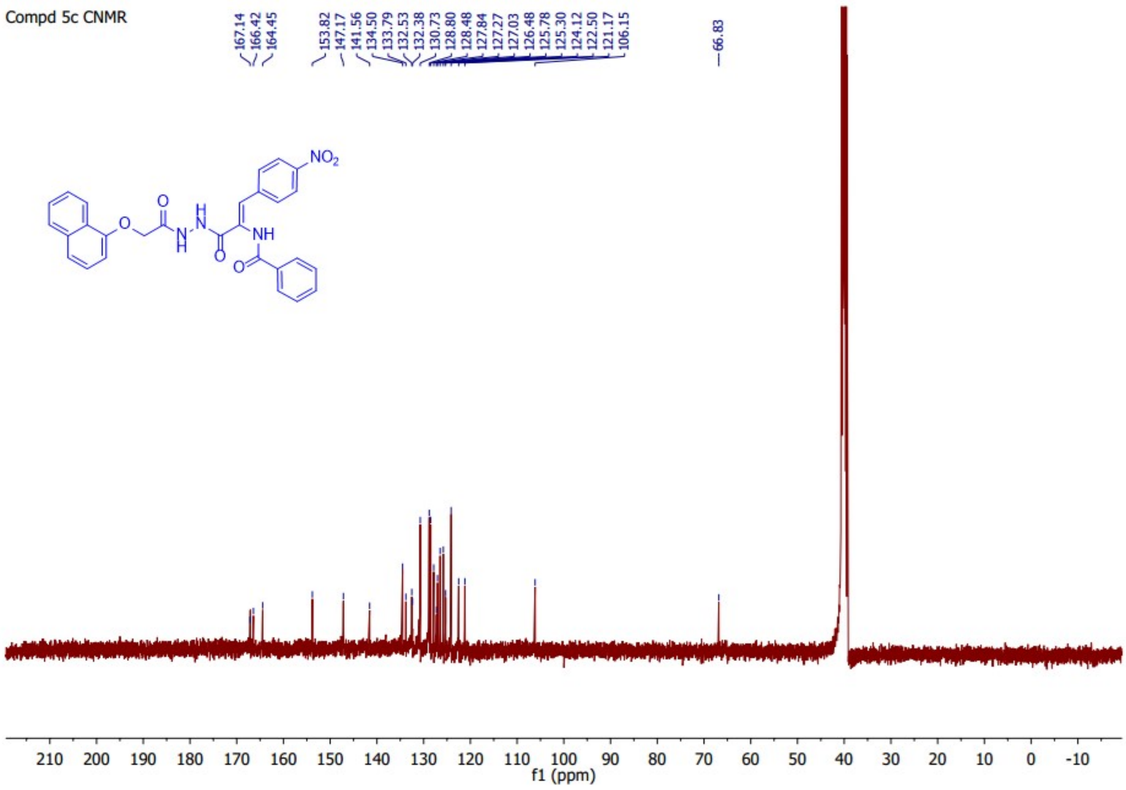

Figure S7: ^{13}C-NMR spectrum of compound **5c**

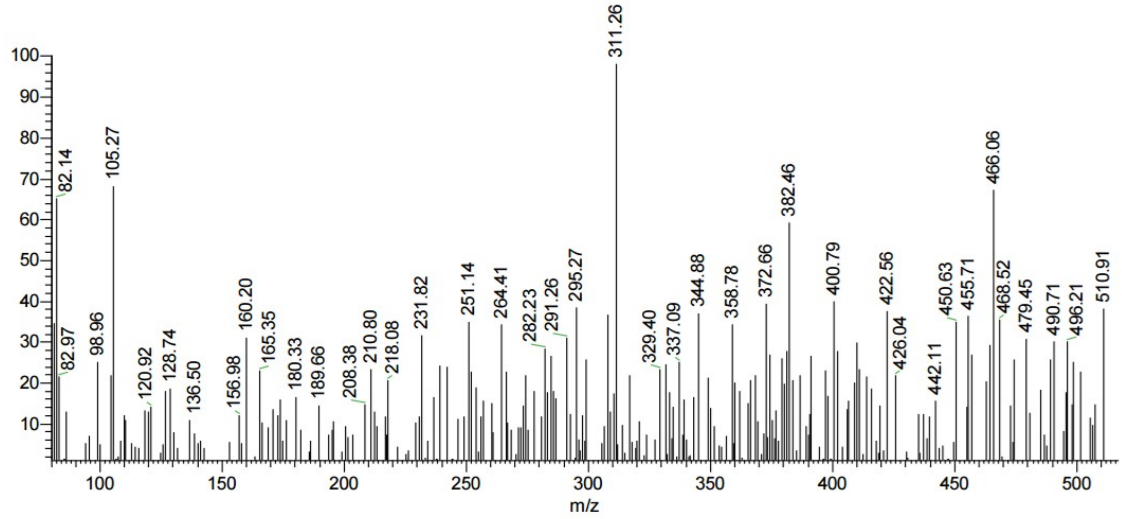

Figure S8: Mass spectrum of compound **5c**

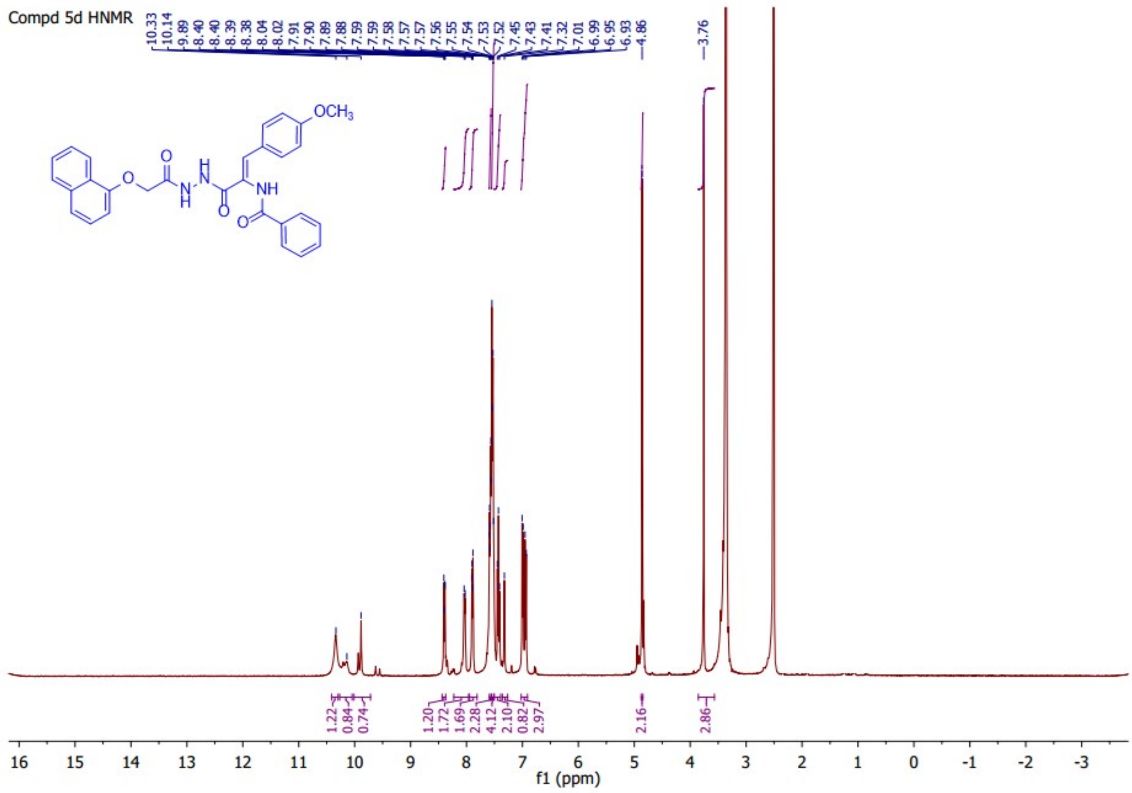

Figure S9: ^{1}H-NMR spectrum of compound **5d**

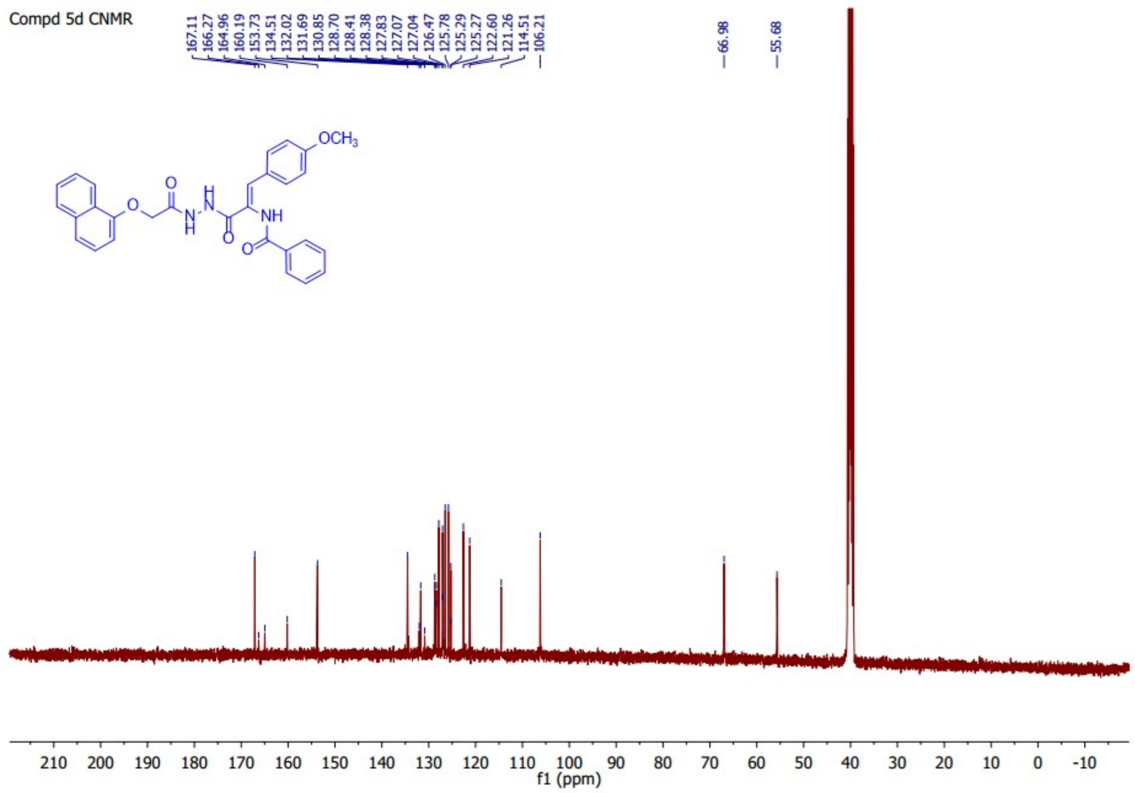

Figure S10: ^{13}C-NMR spectrum of compound **5d**

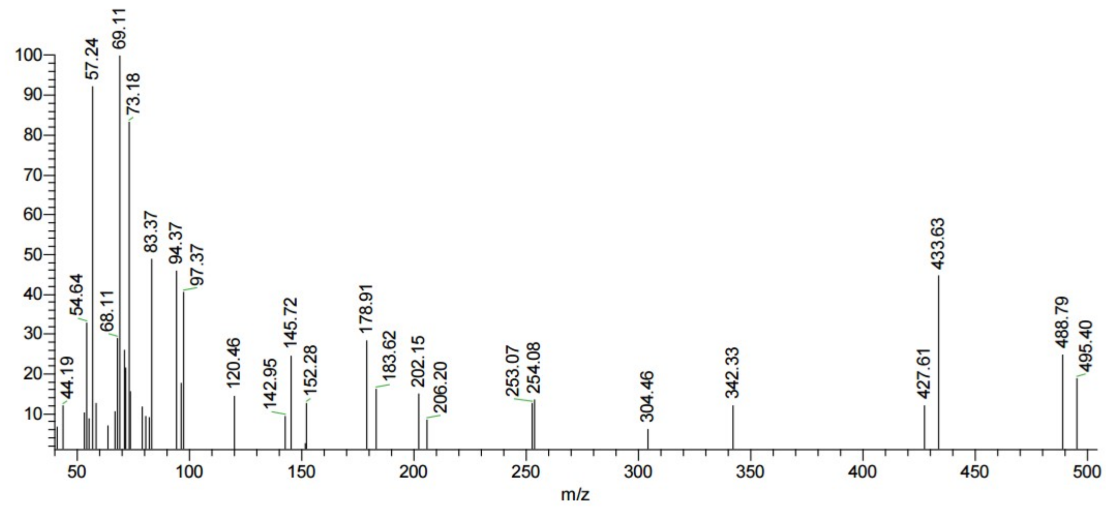

Figure S11: Mass spectrum of compound **5d**

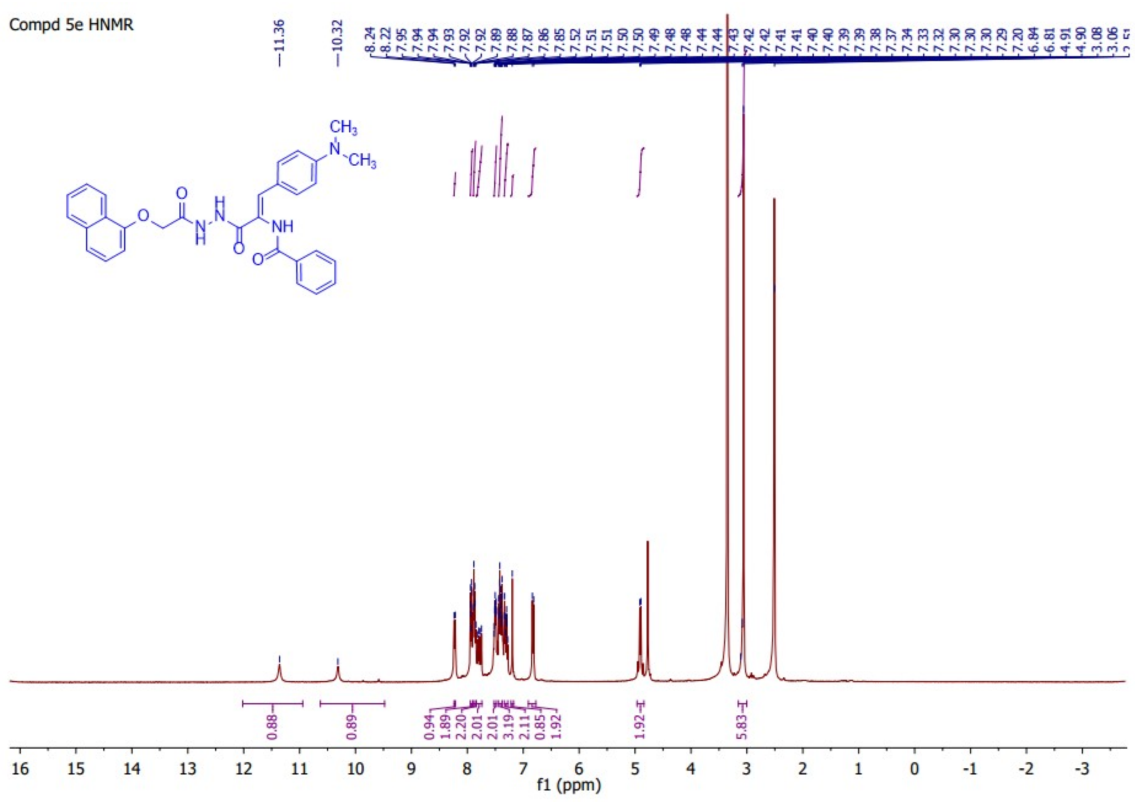

Figure S12: ^{1}H-NMR spectrum of compound **5e**

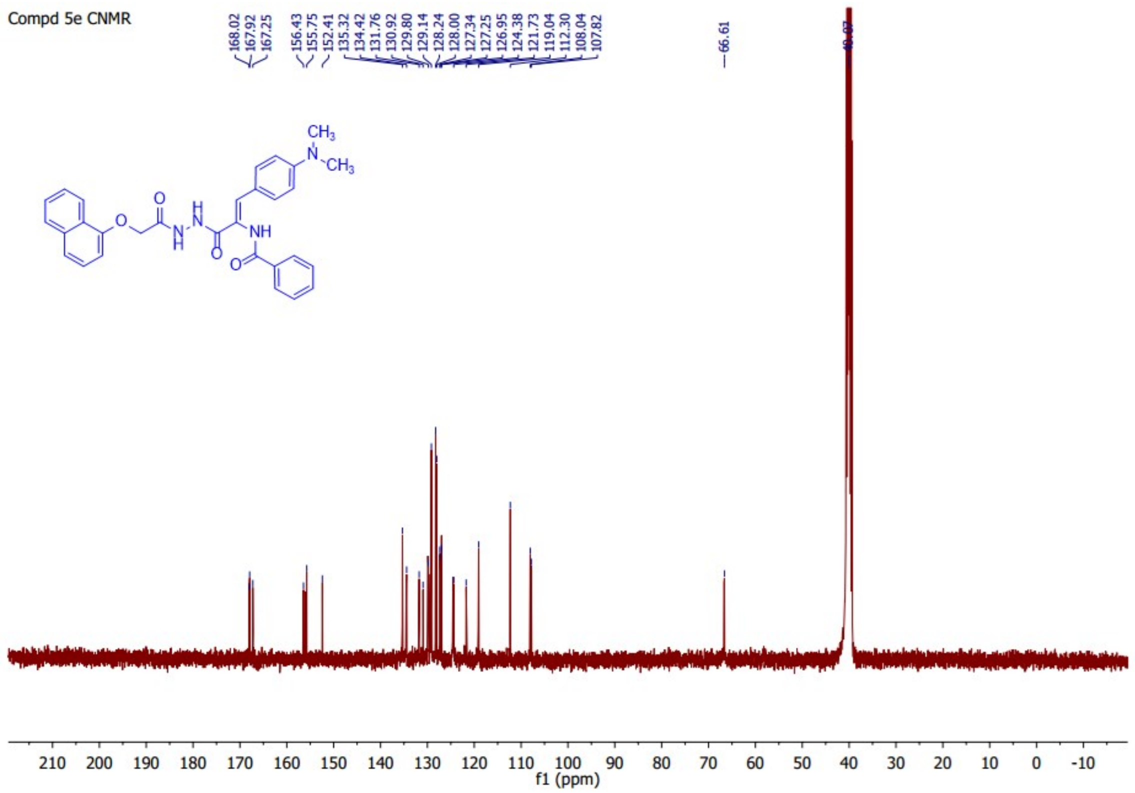

Figure S13: ^{13}C-NMR spectrum of compound **5e**

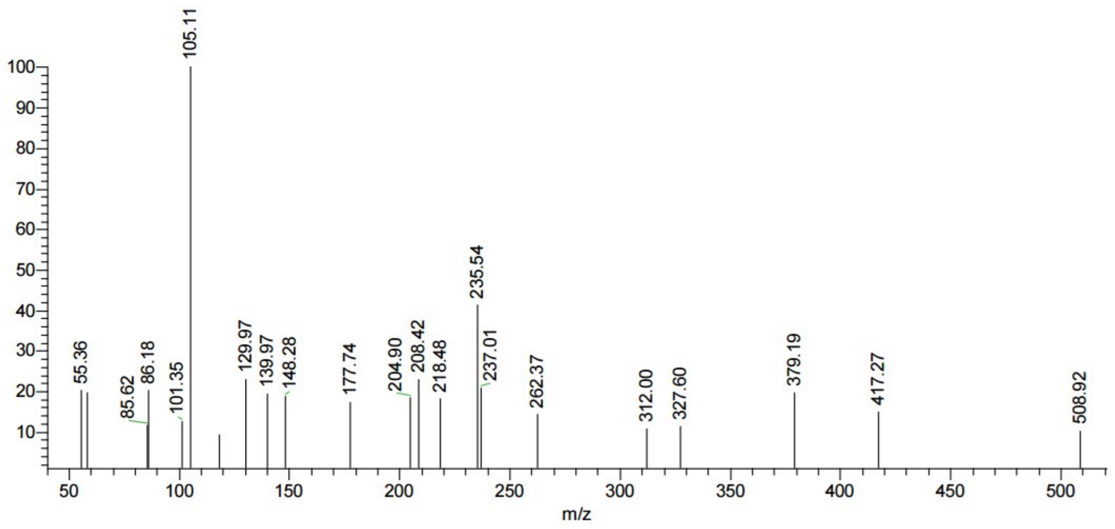

Figure S14: Mass spectrum of compound **5e**

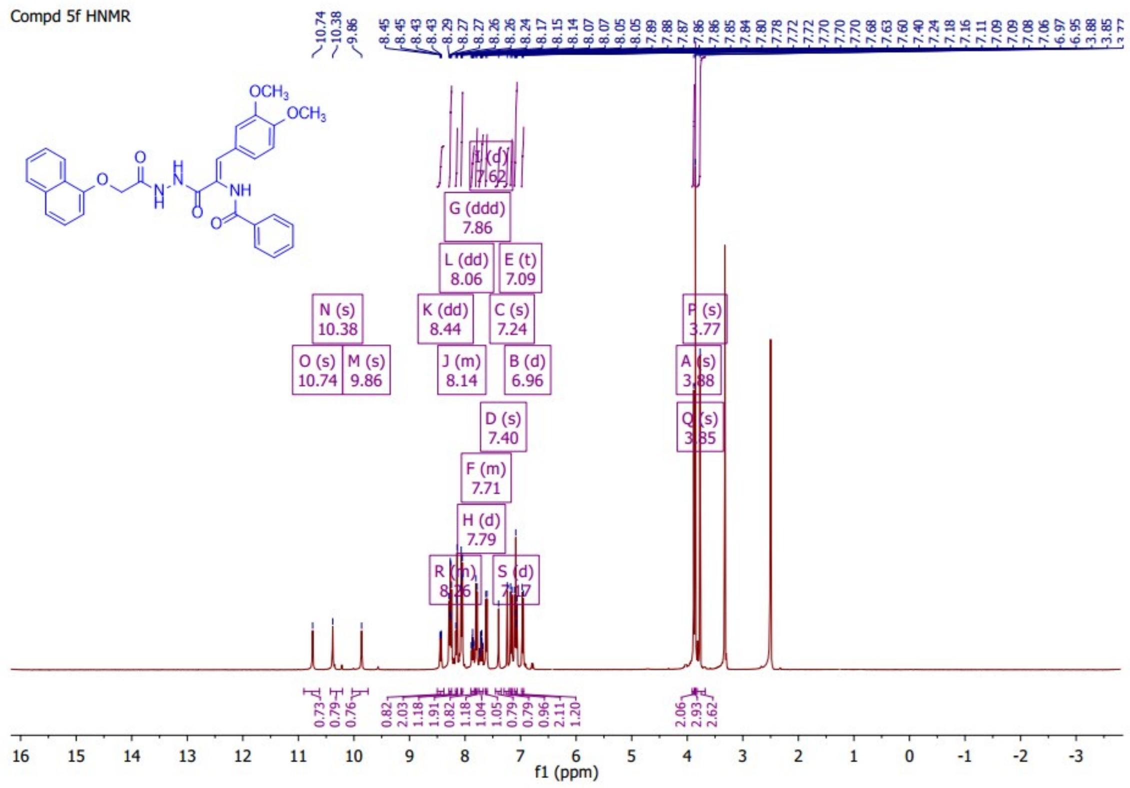

Figure S15: ^{1}H-NMR spectrum of compound **5f**

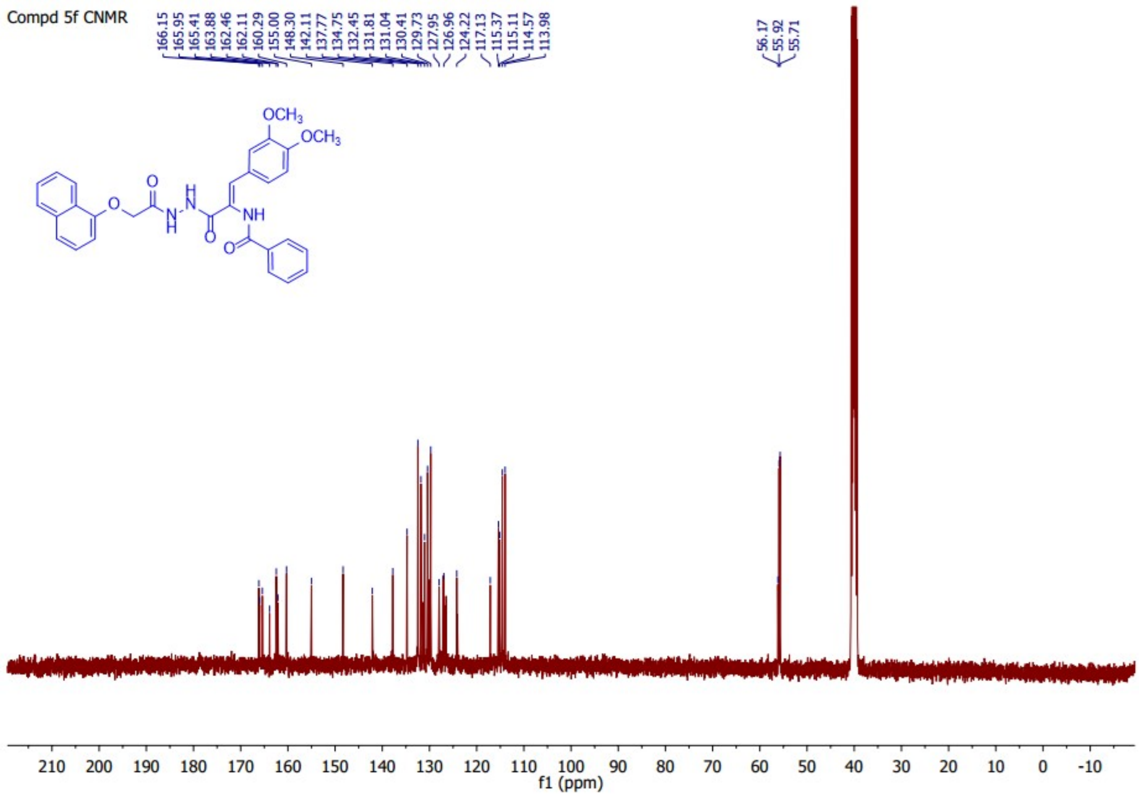

Figure S16: ^{13}C-NMR spectrum of compound **5f**

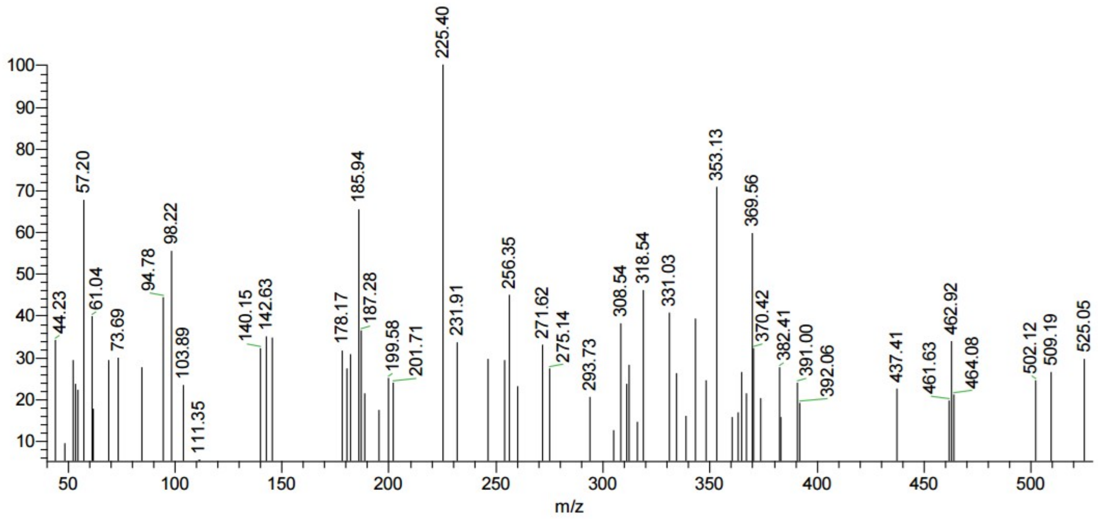

Figure S17: Mass spectrum of compound **5f**

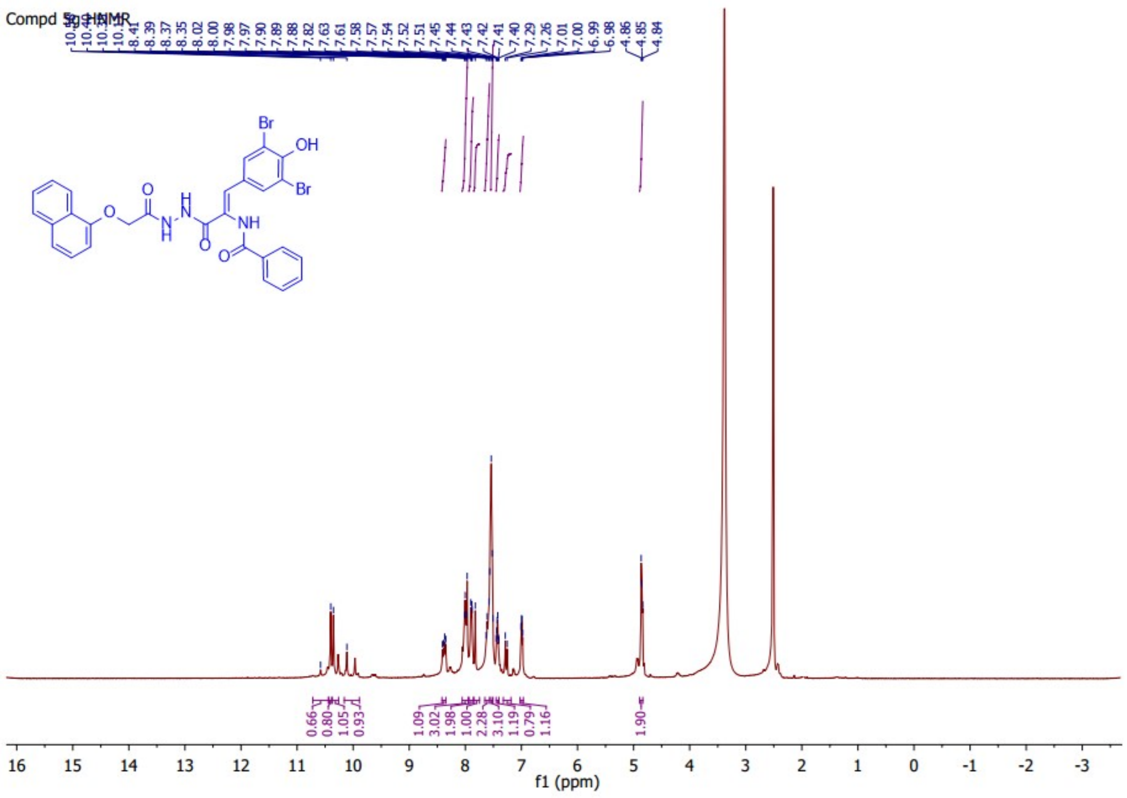

Figure S18: ^{1}H-NMR spectrum of compound **5g**

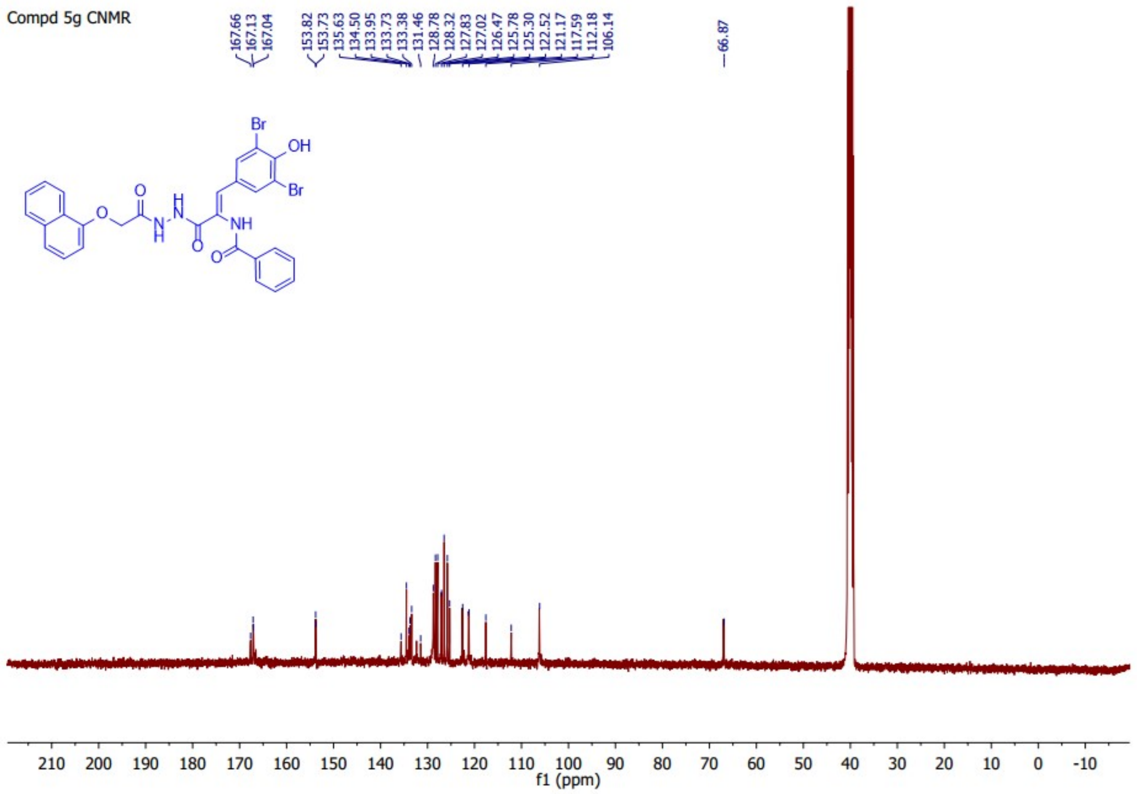

Figure S19: ^{13}C-NMR spectrum of compound **5g**

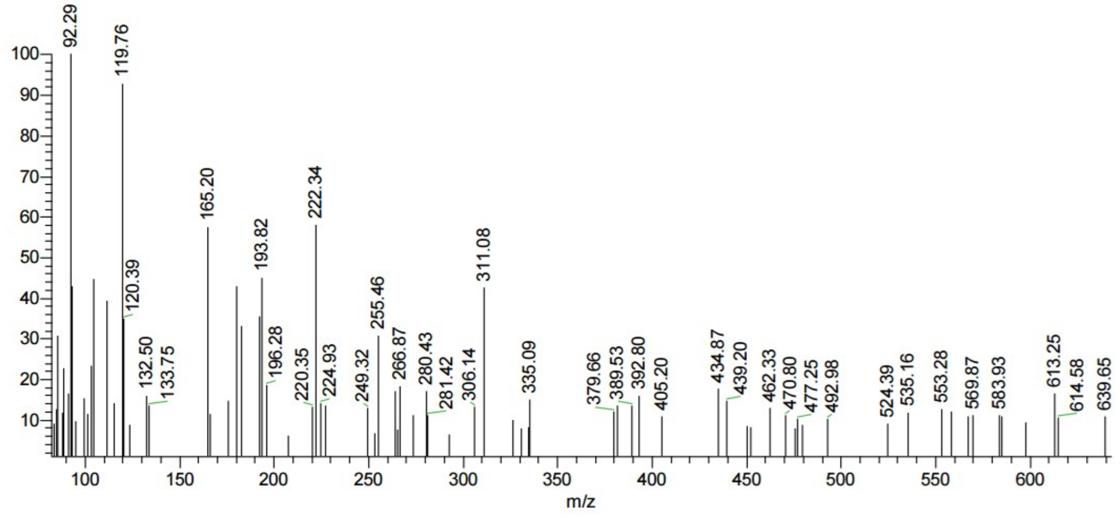

Figure S20: Mass spectrum of compound **5g**

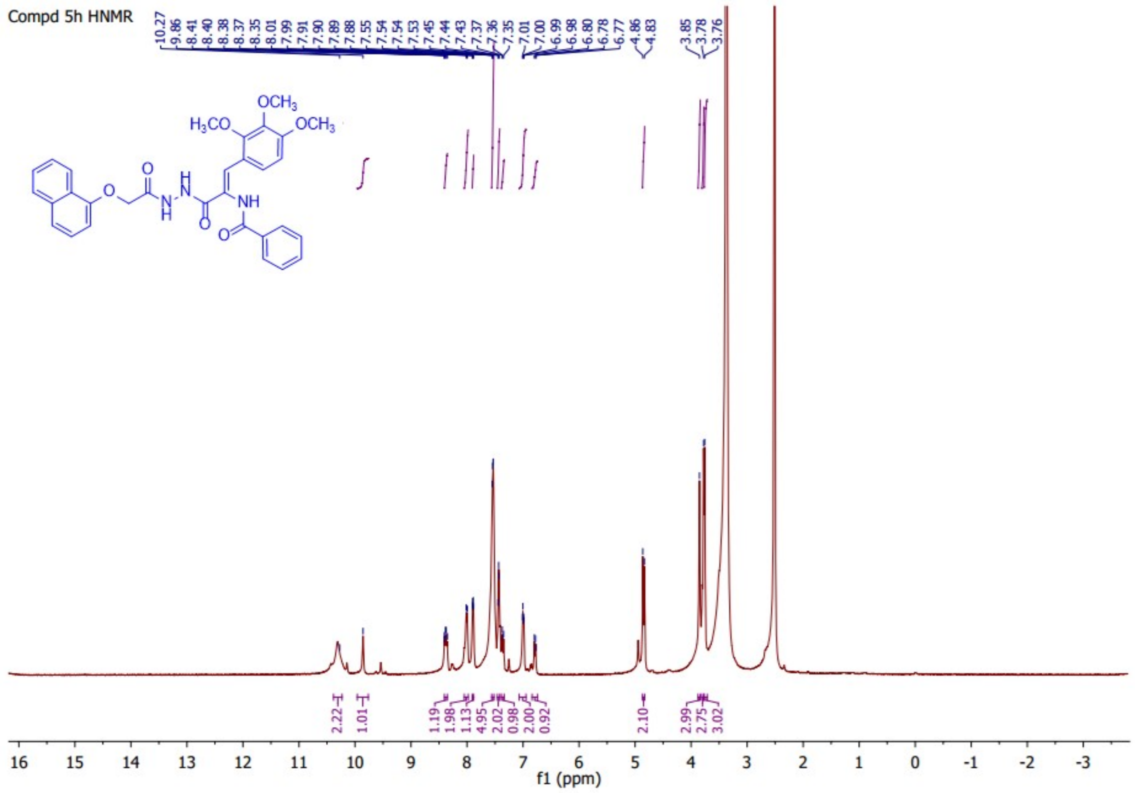

Figure S21: ^{1}H-NMR spectrum of compound **5h**

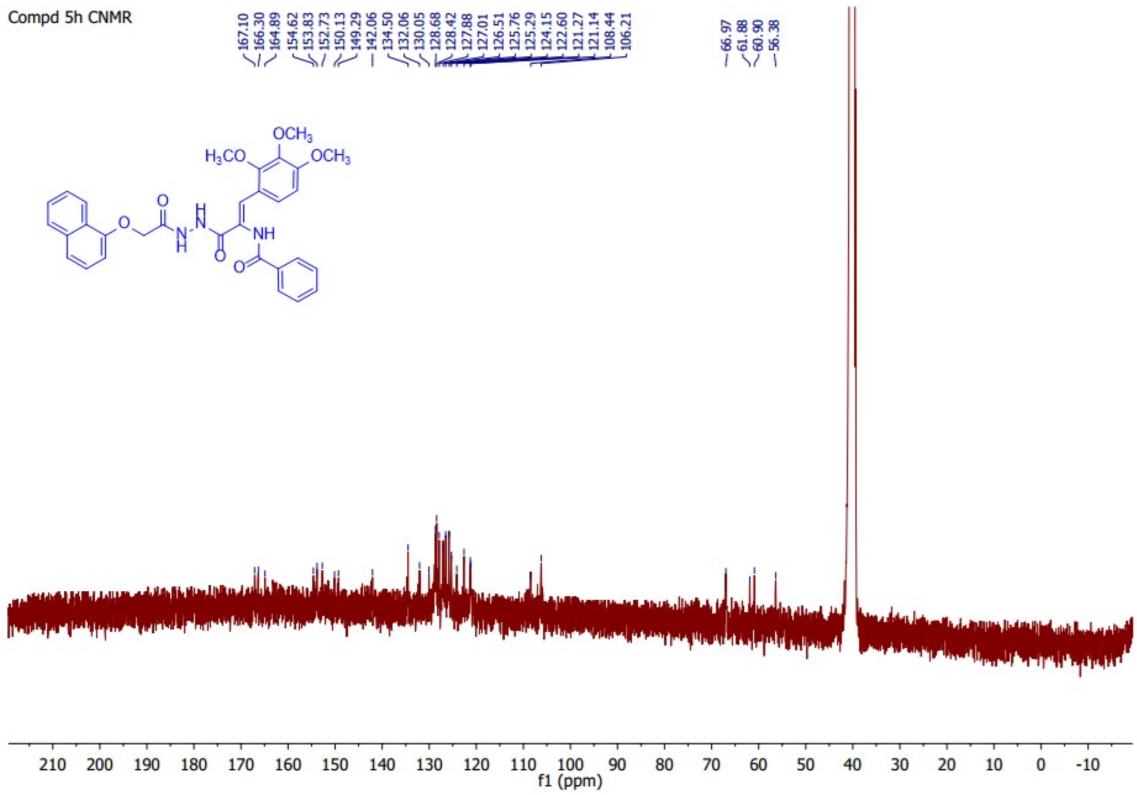

Figure S22: ^{13}C-NMR spectrum of compound **5h**

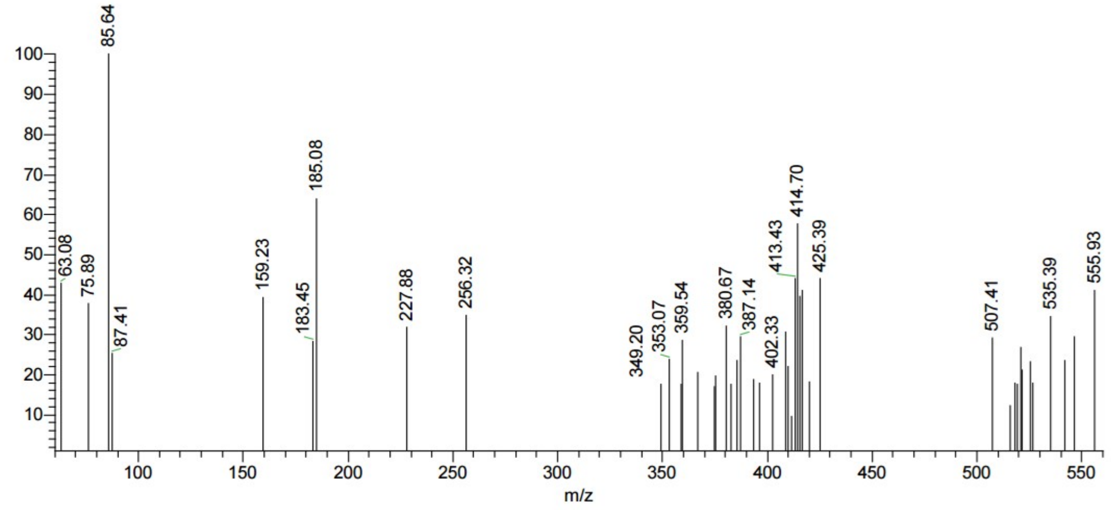

Figure S23: Mass spectrum of compound **5h**

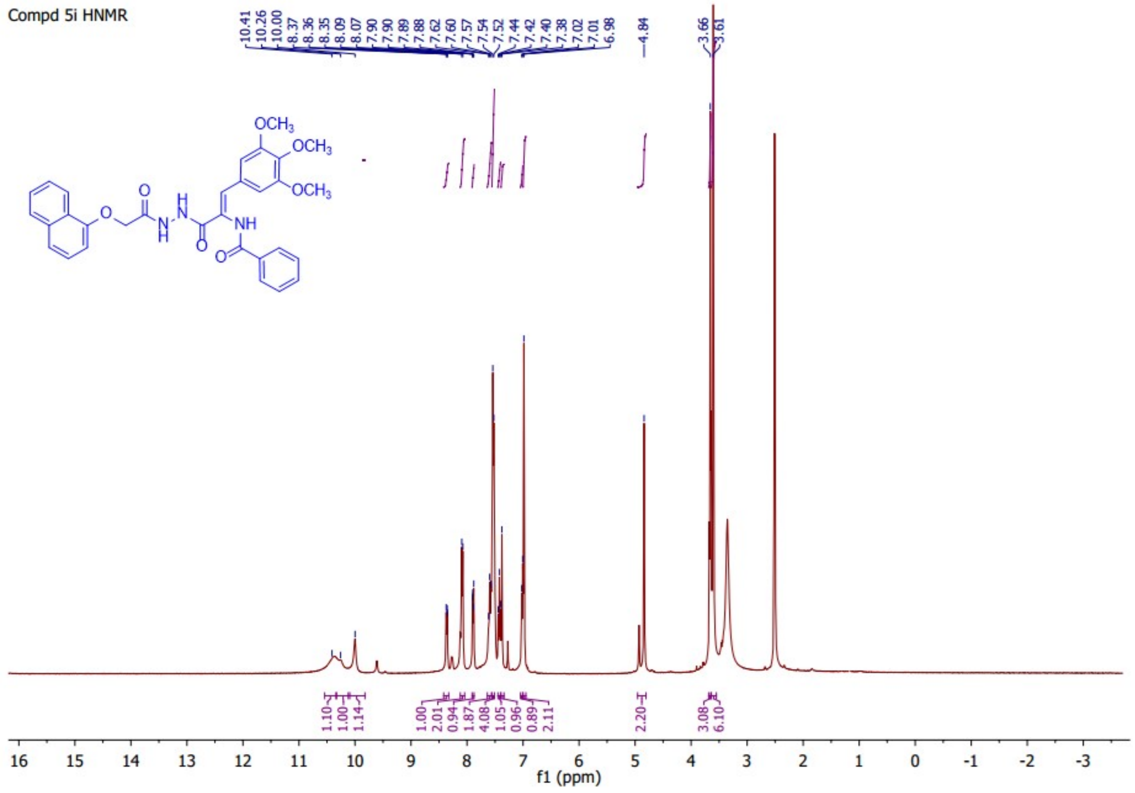

Figure S24: ^{1}H-NMR spectrum of compound **5i**

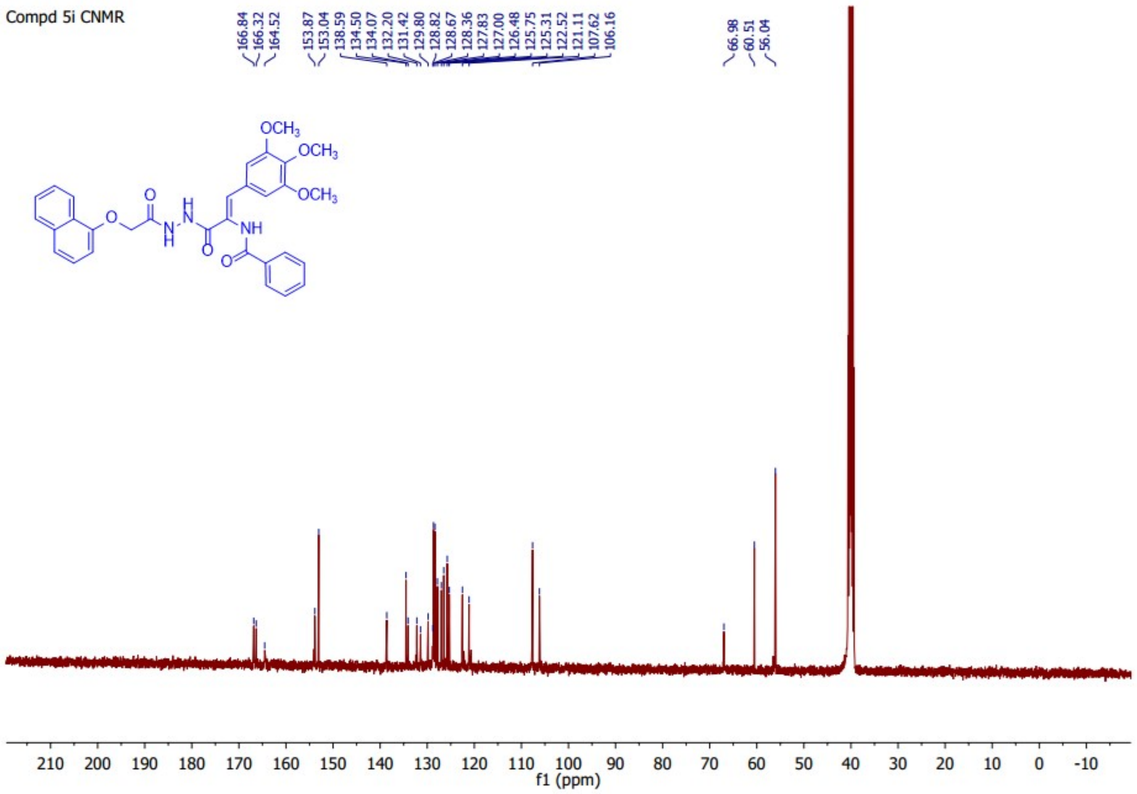

Figure S25: ^{13}C-NMR spectrum of compound **5i**

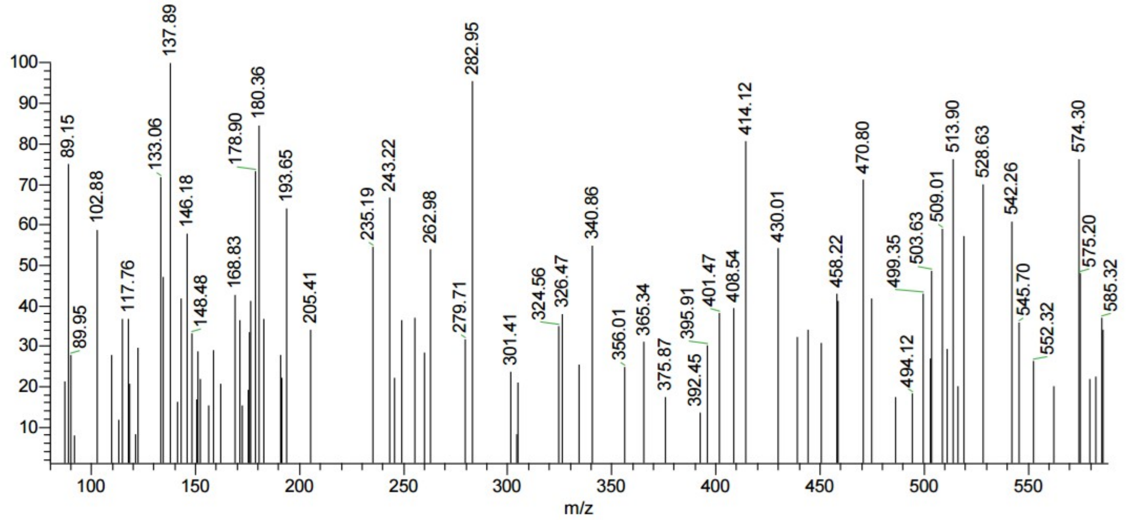

Figure S26: Mass spectrum of compound **5i**

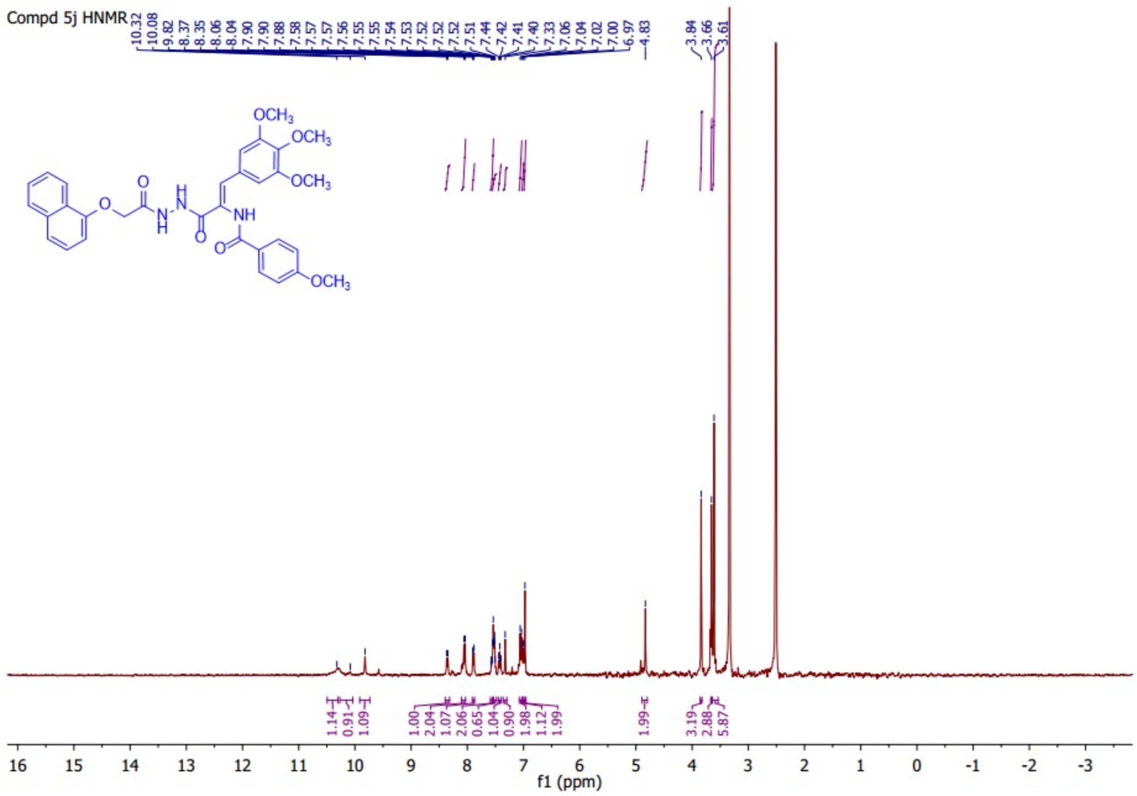

Figure S27: ^{1}H-NMR spectrum of compound **5j**

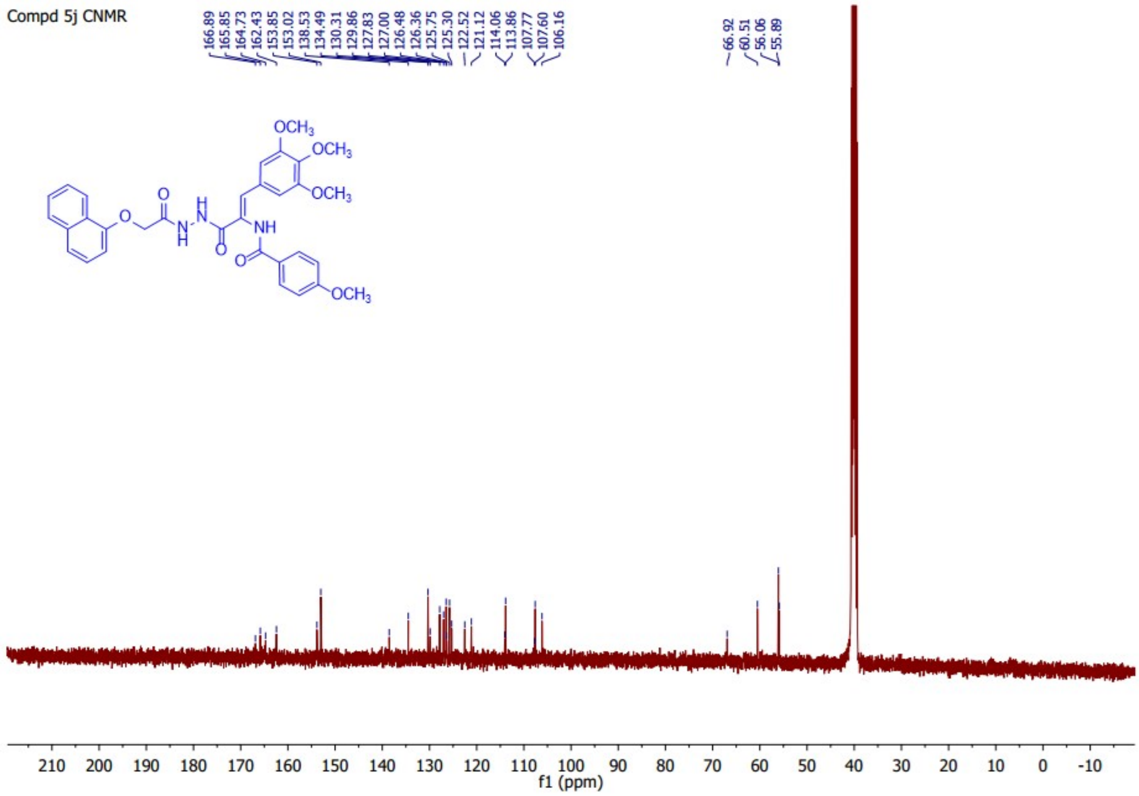

Figure S28: ^{13}C-NMR spectrum of compound **5j**

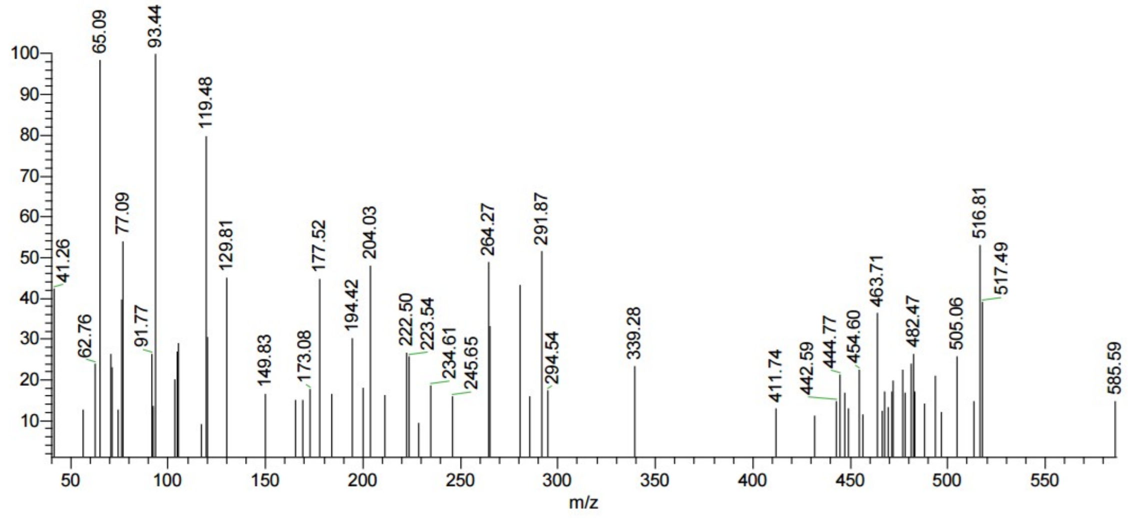

Figure S29: Mass spectrum of compound **5j**

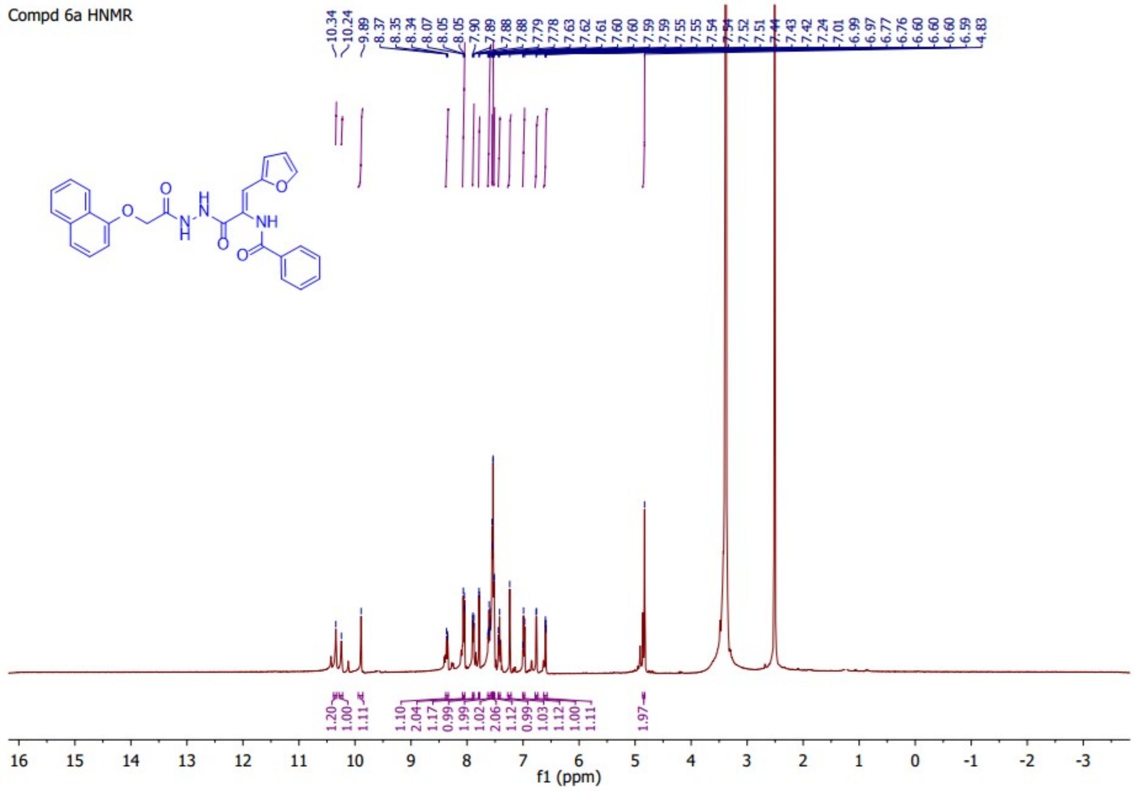

Figure S30: ^{1}H-NMR spectrum of compound **6a**

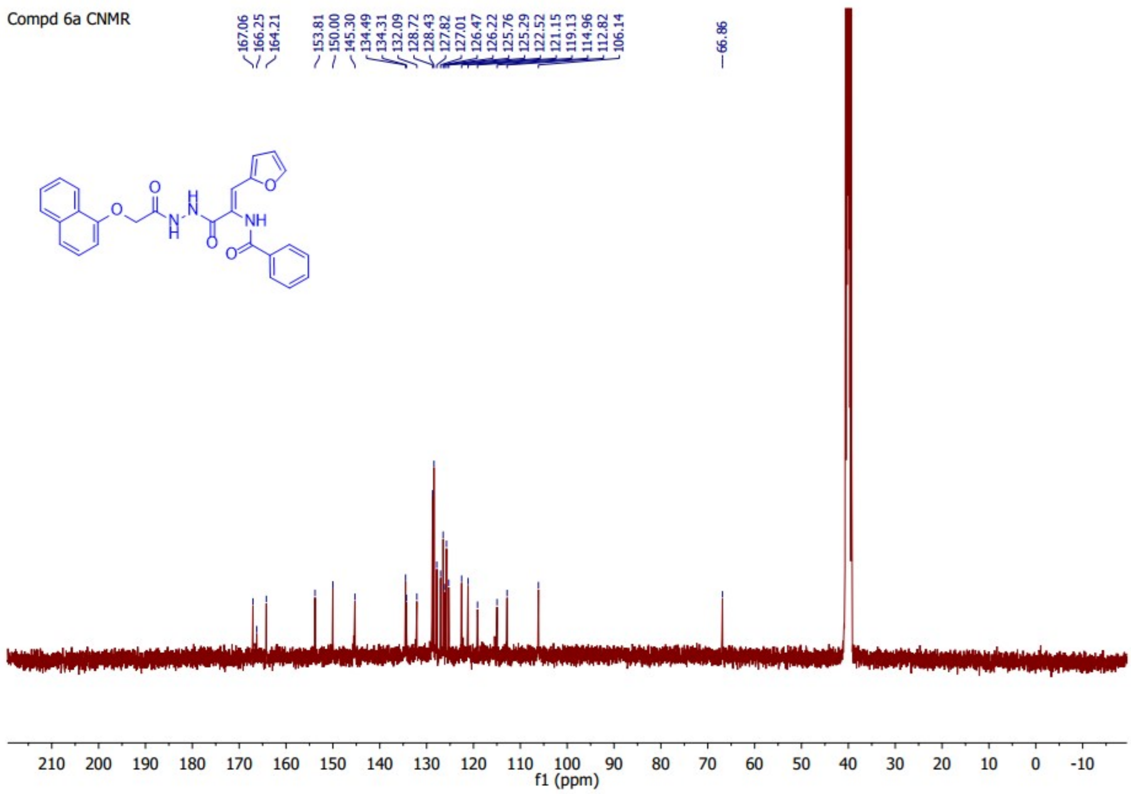

Figure S31: ^{13}C-NMR spectrum of compound **6a**

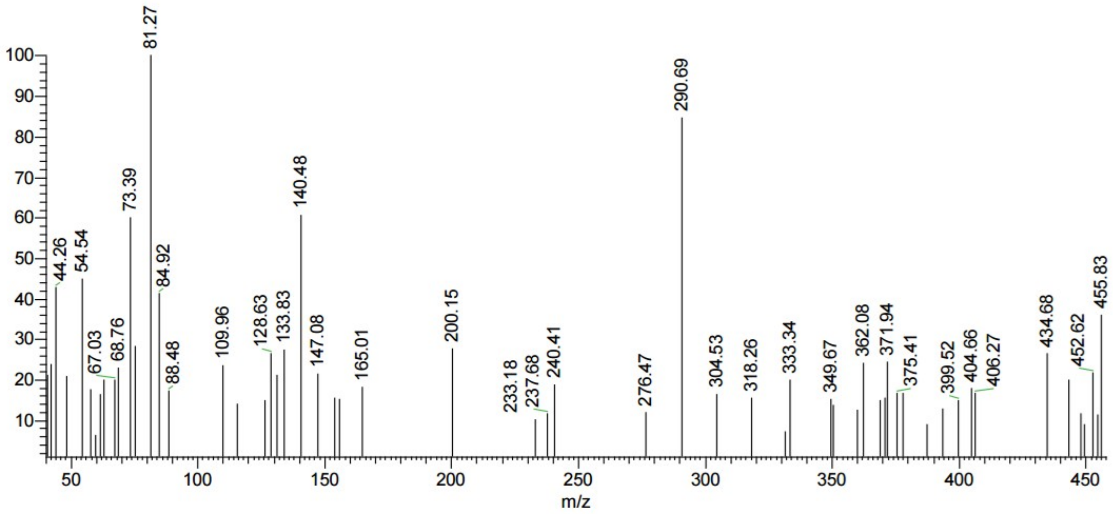

Figure S32: Mass spectrum of compound **6a**

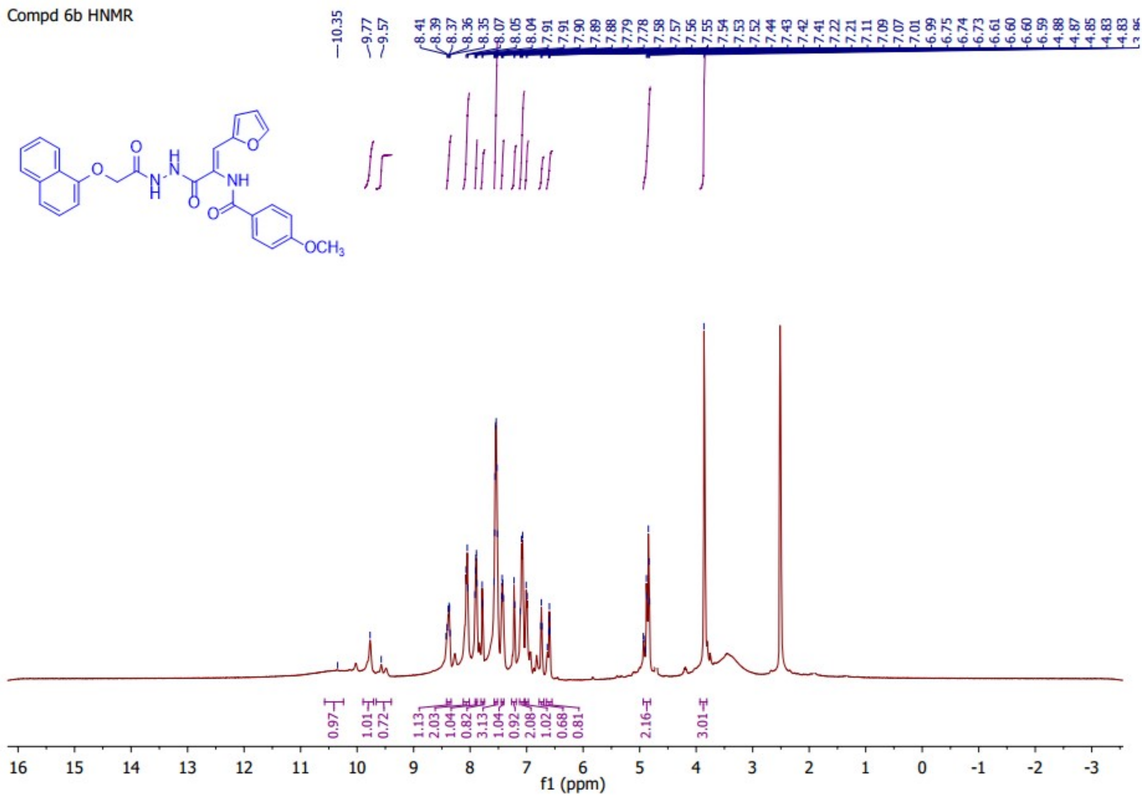

Figure S33: ^{1}H-NMR spectrum of compound **6b**

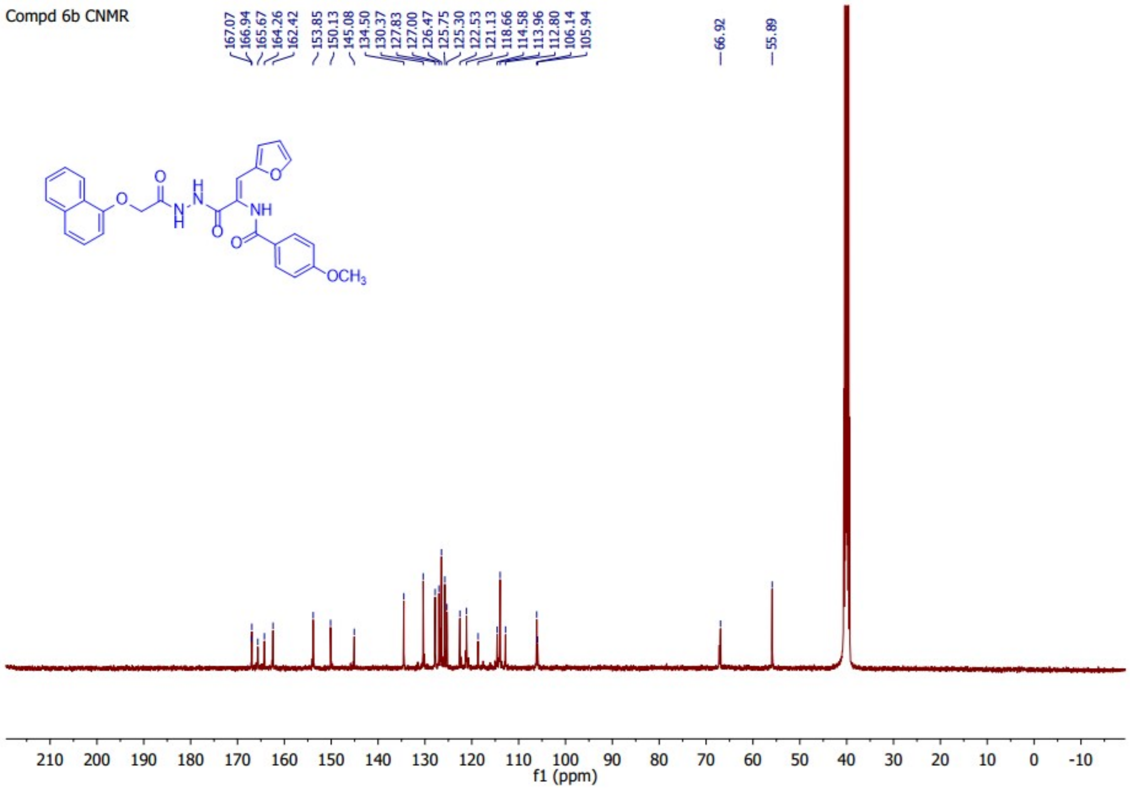

Figure S34: ^{13}C-NMR spectrum of compound **6b**

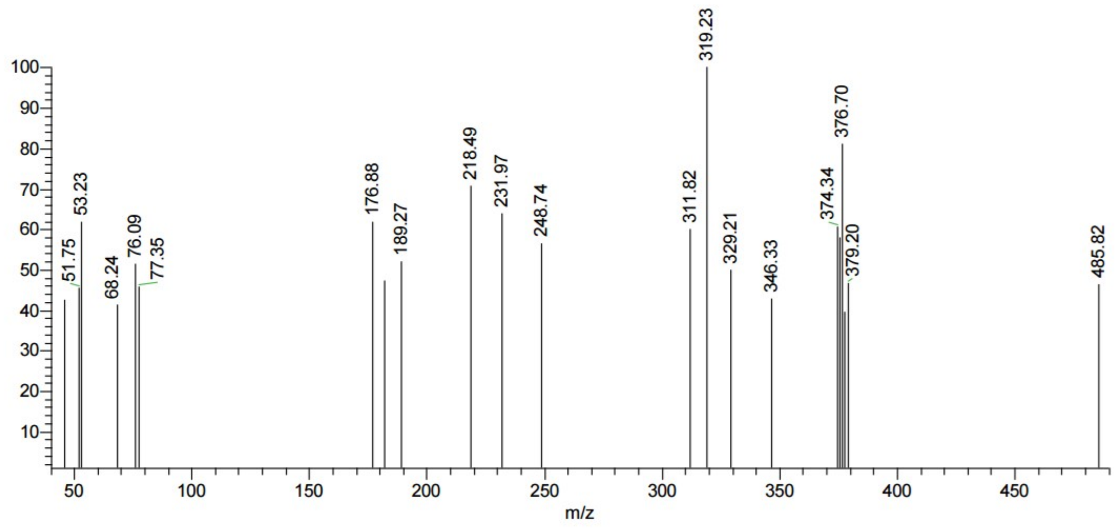

Figure S35: Mass spectrum of compound **6b**

Appendix A

S4.2. Biological Studies

S4.2.1. Cytotoxic activity evaluation

To measure the cytotoxic actvity of the synthesized naphthalen-1-yloxyacetamide tethered acrlamide derivatives **5a-j** and **6a,b** in breast adenocarcinoma (MCF-7) cell line (ATCC Cat. No. HTB-22). Cell viability assay was assessed using MTT assay method. Cells at density of 1 x 10^4 were seeded in a 96-well plate at 37 ^{o}C for 24 h under 5% CO_2. After incubation, the cells were treated with different concentrations of the investigated naphthalen-1-yloxyacetamide tethered acrlamide derivatives **5a-j** and **6a,b** and incubated for 24 h, then 20 µl of MTT solution at 5 mg/mL was applied and incubated for 4 h at 37 °C. Dimethyl sulphoxide (DMSO) in volume of 100 µl was added to each well to dissolve the purple formazan that had formed. The color intensity of the formazan product, which represents the growth condition of the cells, is quantified by using an ELISA plate reader (EXL 800, USA) at 570 nm absorbance. The experimental conditions were carried out with at least three replicates, and the experiments were repeated at least three times.

S4.2.2. Aromatase inhibition Assay

Compounds **5c, 5d, 5e** and Letrozozle were evaluated for their aromatase inhibitory activity according to manufacturer's instructions.

Cloud-Clone Corp.

SEC319Ra 96 Tests
Enzyme-linked Immunosorbent Assay Kit
For Aromatase (ARO)
Organism Species: Rattus norvegicus (Rat)
Instruction manual

FOR IN VITRO AND RESEARCH USE ONLY
NOT FOR USE IN CLINICAL DIAGNOSTIC PROCEDURES

11th Edition (Revised in July, 2013)

[INTENDED USE]

The kit is a sandwich enzyme immunoassay for in vitro quantitative measurement of aromatase in rat serum, plasma, tissue homogenates and other biological fluids.

[REAGENTS AND MATERIALS PROVIDED]

Reagents	Quantity	Reagents	Quantity
Pre-coated, ready to use 96-well strip plate	1	Plate sealer for 96 wells	4
Standard	2	Standard Diluent	1×20mL
Detection Reagent A	1×120μL	Assay Diluent A	1×12mL
Detection Reagent B	1×120μL	Assay Diluent B	1×12mL
TMB Substrate	1×9mL	Stop Solution	1×6mL
Wash Buffer (30 × concentrate)	1×20mL	Instruction manual	1

[MATERIALS REQUIRED BUT NOT SUPPLIED]

1. Microplate reader with 450 ± 10nm filter.
2. Precision single or multi-channel pipettes and disposable tips.

S4.2.3. Cell cycle analysis of compound 5d

Cell cycle analysis in MCF-7 cells was investigated using fluorescent Annexin V-FITC/ PI detection kit (*BioVision* EZCell^{TM} Cell Cycle Analysis Kit Catalog #K920) by flow cytometry assay. MCF-7 cells at a density of 2×10^5 per well were harvested and washed twice in PBS. After that, the cells were incubated at 37 °C and 5% CO_2. The medium was incubated with the tested compound **5d** at the IC_{50} (μM) for 48 h, washed twice in PBS, fixed with 70% ethanol, rinsed again with PBS. Afterward, medium was stained with DNA fluorochrome PI for 15 min at 37 °C. The samples were immediately analyzed using Facs Calibur flow cytometer (Becton and Dickinson, Heidelberg, Germany).

S4.2.4. Fluorochrome Annexin-V/PI assay for compound 5d

Apoptosis in MCF-7 cells was investigated using fluorescent Annexin V-FITC/ PI detection kit (*BioVision* Annexin V-FITC Apoptosis Detection Kit, Catalog #: K101) by flow cytometry assay. MCF-7 cells at a density of 2×10^5 per well were treated with compound **4j** at the IC_{50} (µM) for 48 h, then the cells were harvested and stained with Annexin V-FITC/ PI dye for 15 min in the dark at 37 °C. The samples were immediately analyzed using *FACS Calibur* flow cytometer (Becton and Dickinson, Heidelberg, Germany).

6. ELISA measurements of Bcl-2, Bax and *C*aspase 9

Bcl-2, Bax and *C*aspase 9 activities in MCF-7 *Br Ca* cells were detected in the presence of conjugate **5d** at the IC_{50} concentration (µM). The levels of anti-apoptotic marker Bcl-2, apoptotic markers Bax as well as *C*aspase 9 were assessed using appropriate ELISA kit. The procedure of the used kits was done according to the manufacturer's instructions. Briefly, Cell lysates were prepared from control and MCF-7 cells (2.5 x 10^5/mL) treated with IC_{50} concentration of compound **5d**. Then equal amounts of cell lysates were loaded then probed with specific antibodies. The samples were measured at 450 nm in ROBONEK P2000 ELISA reader. Analysis was confirmed with three different sets of extracts. All experiments were done in triplicates.
